# Talking Dead. New burials from Tron Bon Lei (Alor Island, Indonesia) inform on the evolution of mortuary practices from the terminal Pleistocene to the Holocene in Southeast Asia

**DOI:** 10.1371/journal.pone.0267635

**Published:** 2022-08-24

**Authors:** Sofia C. Samper-Carro, Sue O’Connor, Mahirta, Shimona Kealy, Ceri Shipton

**Affiliations:** 1 Archaeology and Natural History, College of Asia and the Pacific, Australian National University, Canberra, ACT, Australia; 2 ARC Centre of Excellence for Australian Biodiversity and Heritage, Australian National University, Canberra, ACT, Australia; 3 Centre d’Estudis del Patrimoni Arqueologic, Facultat de Lletres, Universitat Autónoma de Barcelona, Bellaterra, Spain; 4 Departemen Arkeologi, Fakultas Ilmu Budaya, Universitas Gadjah Mada, Yogjakarta, Indonesia; 5 Institute of Archaeology, University College London, London, United Kingdom; New York University, UNITED STATES

## Abstract

Burial elaborations are a human behaviour that, in recent contexts can inform on social diversification, belief systems, and the introduction of new practices resulting from migration or cultural transmission. The study of mortuary practices in Mainland and Island Southeast Asia has revealed complex and diverse treatments of the deceased. This paper contributes to this topic with the description of three new burials excavated in Tron Bon Lei (Alor Island, Indonesia) dated to 7.5, 10, and 12 kya cal BP. In addition to the bioskeletal profiles and palaeohealth observations, we propose the adoption of archaeothanatological methods to characterise burial types in the region. Through the analysis of skeletal element representation, body position, articulation, and grave associations, we provide an example of a holistic approach to mortuary treatments in the Lesser Sunda Islands. Our results provide significant new data for understanding the evolution and diversification of burial practices in Southeast Asia, contributing to a growing body of literature describing prehistoric socio-cultural behaviour in this region.

## Introduction

The study of burial practices from the Pleistocene onwards has garnered significant interest among researchers over the last thirty years, with the development of field anthropology or archaeothanatology leading to the reporting of mortuary practices from a holistic point of view [1–6]. By including observations on body positioning, articulation sequences and associated grave goods, this approach to the study of mortuary treatments offers the opportunity to look at burial practice as a cultural entity (e.g. 5). The combination of bioarchaeology, bioanthropology, and archaeothanatology provides a complete picture of the living conditions of the deceased, as well as describing the history of events from peri-mortem to inhumation.

**Fig 1:**
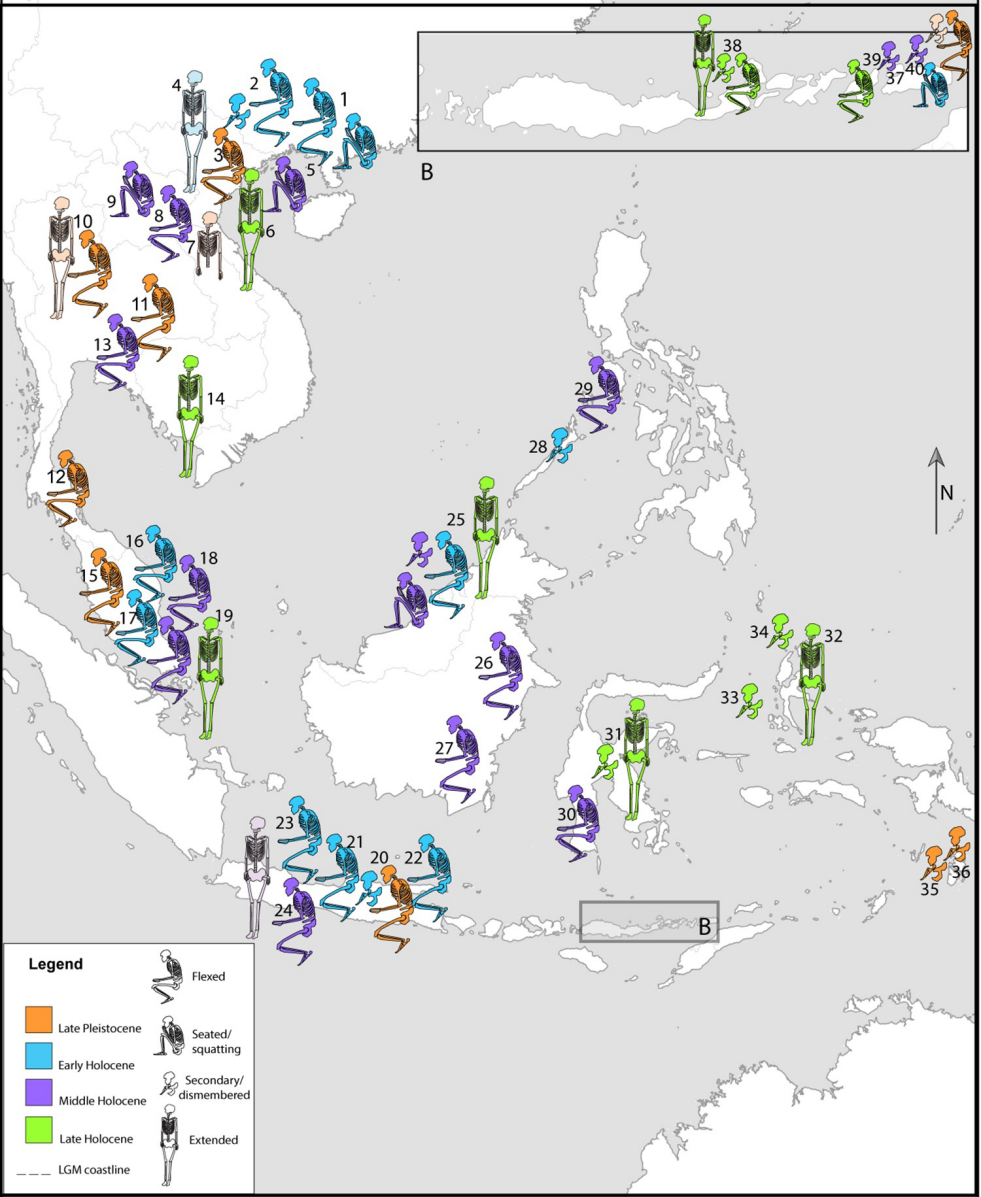
Sites map. Geographic distribution of sites with human burials described in the text. For site numbers, refer to S1 Table. Basemap made with Natural Earth. Free vector and raster map data @ naturalearthdata.com.

The use of archaeothanatology approaches in Mainland (MSEA) and Island Southeast Asia (ISEA) has permitted an assessment of variations in mortuary practices as a means to discuss population replacements, human migration pathways and different aspects of social behaviour [e.g. 3, 7]. Although publications explicitly focusing on these approaches are scarce, the description of burial treatments and bioskeletal profiles of the interred individuals, have been used to review the mortuary practices in MSEA and ISEA from the Pleistocene onwards (Fig 1; S1 Table). Regional descriptions of the evidence available on burial treatments in MSEA and ISEA is included in the S1 File.

The review of burial practices from the Late Pleistocene to recent times in Mainland and Island Southeast Asia illustrates the complexity and diversity of mortuary treatments in the region. From changes in body positioning and skeletal element treatment, to the presence/absence of grave associations in the form of grave goods or manuports, Southeast Asian sites offer a panoply of social expressions related to the deposition of the deceased.

This paper contributes additional information on mortuary practices in the region by introducing two new human burials documented in Tron Bon Lei (Alor Island, Indonesia), complemented by the study of the postcranial skeleton of the individual for which craniometric data and grave goods have been previously published [8, 9]. The analysis presented includes the anatomical description, bioskeletal profiling and funerary archaeology evidence.

## Material and methods

All necessary permits were obtained for the described study, which complied with all relevant regulations. The material described was excavated under a RISTEK Foreign Research Permit granted by the Indonesian government (O’Connor 1304/FRP/SM/V/2014 & 3024/FRP/E5/Dit.KI/IX/2017). Following excavation, the material was transported to the Archaeology Department at the Universitas Gadjah Mada (UGM; Yogyakarta, Indonesia). Upon signing of a Material Transfer Agreement for non-destructive analysis between the Universitas Gadjah Mada and the Australian National University (ANU), the material was imported under a permit to import biological products granted by the Australian Government, Department of Agriculture, Water and Environment (0004271052), and deposited at the quarantine accredited site (room 3.127) in Coombs Building (Department of Archaeology and Natural History, ANU), where it is currently kept. The analysis of the remains followed the guidelines established in the Code of Ethics of the American Association of Physical Anthropologists (2003). The material will be returned to the UGM (Archaeology Department) and stored with the rest of the archaeological material recovered from Tron Bon Lei upon publication of the results. No catalogue accession numbers have yet been assigned to the material as it has not been deposited in a museum.

### Tron Bon Lei: Previous research and new excavations

**Fig 2:**
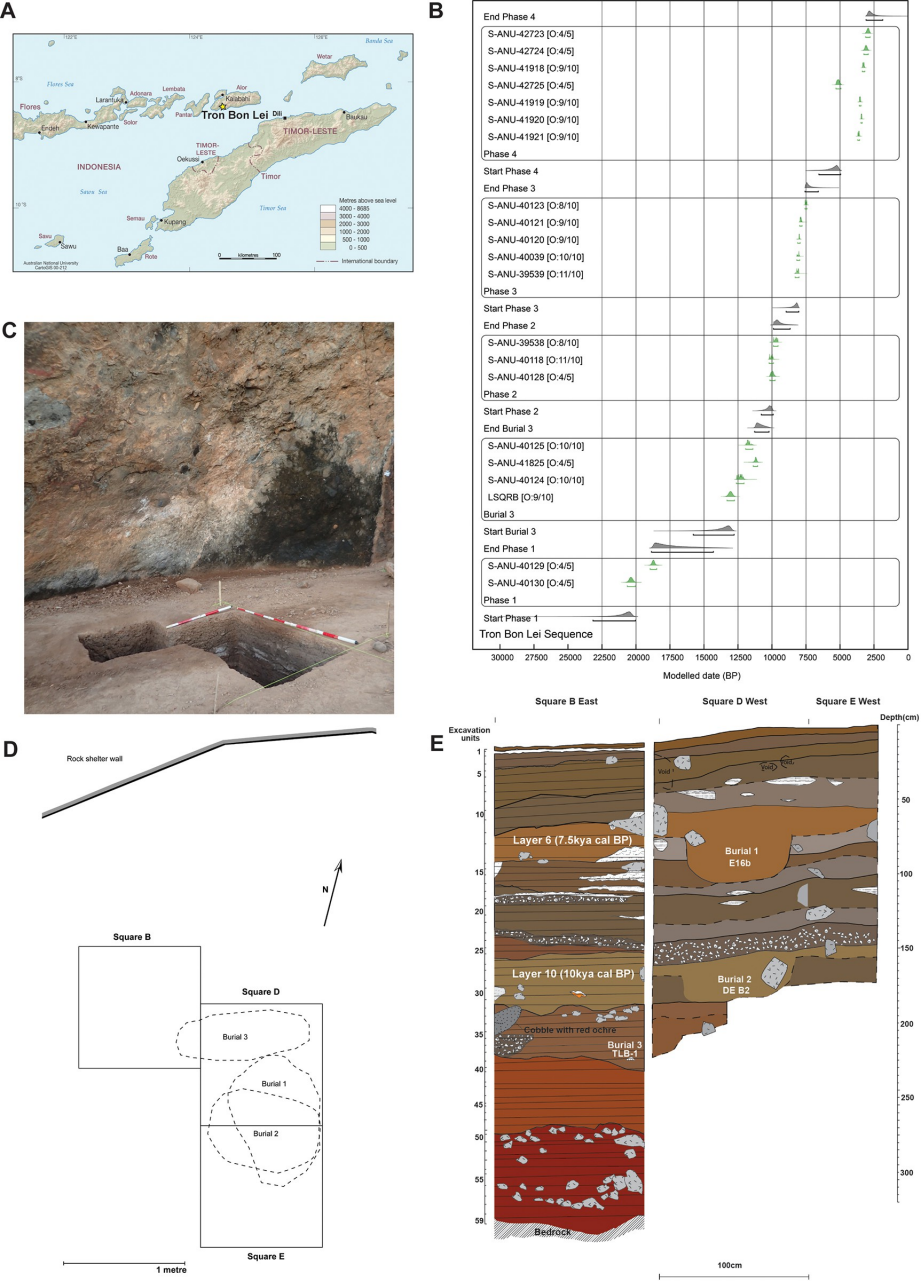
Location, chronology and stratigraphy of Tron Bon Lei. A) Location of Tron Bon Lei on the south coast of Alor. Map reproduced with the permission of the CartoGIS Services, Scholarly Information Services, The Australian National University; B) Bayesian model of dates for Tron Bon Lei, square B. Modelled in OxCal v.4.4. [13] with the Marine20 [14] calibration curve for marine shell samples and a U(0,50) mixed calibration curve with IntCal20 [15] and SHCal20 [16], as recommended for the Inter-Tropical Convergence Zone [16, 17]; C) General view of squares B, D and E; D) Schematic drawing illustrating the morphology of the grave cuts for each of the burials from Tron Bon Lei; E) Combined stratigraphy of squares B, D and E.

Tron Bon Lei is comprised of two adjoining shelters located within a series of shelters in a volcanic ridge which runs along the south coast of Alor, near Lerabain village (Fig 2A). Situated ca. 35m asl and 160m inland, excavations in 2014 documented a large amount of vertebrate and invertebrate fauna, lithics, shell ornaments, and fish-hooks which occurred as grave goods [8, 10, 11]. Human remains were documented in two of the test pits excavated in the two adjoining shelters in 2014 (squares B and C) and the cranial elements have been described elsewhere [9]. The cranial remains from square C (TLC-1) were dated using OSL to c.17kya BP, constituting the earliest human remains thus far identified in the Lesser Sunda Islands [9], and together with the 25-16kya remains from Sulawesi [12], among the earliest for the Wallacean region.

A complete chronometric sequence from the original excavations in Tron Bon Lei identified four phases of human occupations: Late Holocene (c.2.7–5.4kya cal BP), middle Holocene (c.7.4–8.2kya cal BP), early Holocene-terminal Pleistocene (c.9.4–12.7kya cal BP), and Late Pleistocene (c.17.4-21kya cal BP) (Fig 2B).

The analysis of obsidian artefacts from the original Tron Bon Lei excavation indicates three distinctive sources, as well as a specialised technology [18, 19]. One distinctive obsidian type, Group 1 which has also been recorded in nearby Timor-Leste and Kisar island, appears in the sequence only after c.12kya cal BP and is believed to result from maritime transport from a source on a different island [18]. Tron Bon Lei remained a significant location for the groups inhabiting the area until the late Holocene, as suggested by the presence of red pigment rock art on the walls of the rockshelter, with some of these motifs interpreted as compatible with motif repertoire of the Austronesian Painting Tradition, dated within the last 3,000 years [20, 21].

The zooarchaeological analysis of the vertebrate assemblage documented in square B indicates extensive exploitation of marine resources, with changes in the carnivore/herbivore fish ratio and fish size from the Pleistocene to the Holocene layers [9, 10]. Nevertheless, the isotopic signature identified in the human remains from Tron Bon Lei suggest a mixed/terrestrial diet, likely based on the consumption of plant foods, with presumably a larger input of marine protein resources in the Pleistocene individuals, especially for TLC-1 [22].

In 2018, an Australian-Indonesian research team returned to Tron Bon Lei to complete the excavation and recovery of the initial human remains documented in 2014 in square B (Fig 2C and 2D). The original 1x1 test pit was extended to the southeast (Square D) to recover the postcranial remains from the TLB-1 skeleton [9]. Excavation proceeded by stratigraphic context, with similar contexts grouped together into layers and thicker contexts divided into 5cm spits.

At around 80cm depth in square D (spit 14), a concentration of waterworn cobbles and igneous rocks preceded the documentation of a grave cut in spit 16 (E16b; 100-105cm depth), which prompted the extension of a further 1 m^2^ test pit to the south (square E) to record the complete extent of the grave cut (Fig 2C and 2E). This burial (E16b) was excavated into lower layers from layer 6, dated to 7.4–7.6kya cal BP (Fig 2B and 2E; S2 Table). The burial fill consists of grey sandy silt, with frequent shell and obsidian flakes in the upper part, and charcoal fragments in the lower part, contrasting with dark grey/brown sediment from outside the grave cut. The grave cut had an oval shape, with a sharp slope on the upper part of the burial becoming imperceptible at the base (Fig 2D).

After recording and recovery of the human remains in E16b, excavation continued to a depth of 1.60m where another rock and cobble accumulation was identified, preceding the identification of a further grave cut (DE B2, 165-175cm depth). This second burial appeared excavated from the original layer 10, dated to 9.6–10.2kya cal BP (Fig 2B and 2E; S2 Table). The burial fill is grey, darker in colour and more homogenous than the surrounding sediment. Upon removal of the rocks on the top of the burial, large pieces of charcoal were recorded, present throughout the grave cut, with large *Turbo* shells towards the base. The grave cut was roughly circular in shape.

Finally, at a depth of ca. 2.3m (layer 11, 10.1–12.6kya cal BP) the original burial from square B (TLB-1) was located (extending into square D) and excavated [8, 9]. No clear grave cut was observed. However, the burial appeared in a pocket of orange-brown silty sand sediment, surrounded by a compacted darker brown silty clay that corresponds to the sedimentary matrix of layer 12 (ca.18.5-19kya cal BP), suggesting a shallow grave cut from layer 11 which was dug into the layer below, layer 12 [8, 9]. The dates obtained from the burial context and a direct date on a tooth support this interpretation.

TLB-1 individual is dated to c.11.1–12.6kya cal BP (Fig 2B; S2 Table), based on ages obtained from charcoal samples recovered inside the eye socket (S-ANU40125; 10,140±45 BP) and below the cranium (S-ANU 40124; 10,445±50 BP), as well as a direct date (S-ANU 41825; 10,230±30) on one of the shell fish-hooks placed at the neck of the individual [8, 9]. A direct U-Th age for TLB-1 was also obtained from three dentine samples (LSQRB-A, LSQRB-B, and LSQRB-C) on a single tooth with an average corrected age of 13,060±130 BP [9] (S2 Table). While laboratory analysis of the U-Th ages did not detect significant U uptake or loss in the tooth [9], the presence of calcite precipitates in the deposit suggest this system was open to water movement since deposition, which along with the disparity between the U-Th date and the radiocarbon dates, in particular the direct date for the grave goods, strongly suggest that the U-Th estimates are too old for the date of the burial. We therefore consider the direct date on the fish-hook grave good as the more reliable estimate for the age of the burial, bracketed somewhat by the dates from the associated charcoal samples.

### Reconstruction and recording of the human remains

Most of the skeletal elements from the burials were plotted *in situ* with a total station, photographed and prepared for transportation to be stored at the Universitas Gadjah Mada (UGM, Yogjakarta, Indonesia). Human remains were bagged individually and identified to skeletal element when possible. In 2020, the material was transported to the Australian National University (ANU) for further treatment and analysis.

Skeletal elements were cleaned with a dry brush, and those elements with good preservation were further cleaned with ethanol and cotton swabs. Fragmented skeletal elements displaying dry breakage were refitted with masking tape and reconstructed using Paraloid^TM^ B-72 diluted in acetone at 20% Paraloid, following recommendations for the use of Paraloid adhesive for reconstruction purposes [23]. Only those fragments where clear conjoins were identified were physically reconstructed.

Skeletal elements were photographed using a Canon EOS 5D camera with macro lens SIGMA AF 105mm. Bones were measured using digital callipers (Kincrome model K11100, 0.01mm resolution), and an osteological measuring board. GIS data, photographs, and documentation from the excavation of the burials were processed in ArcMap 10.8.1 (ESRI) to create burial profiles and maps. Recording of physical anthropology, funerary archaeology, and contextual information was conducted in HumanOS software [24]. This application allows the recording of burial inventories, including the context of the burial and measurements, as well as data to perform mortuary analysis.

### Bone preservation

To assess the effect of post-mortem and post-depositional processes on the skeletal elements, weathering stages, breakage patterns and calcareous coating were recorded. Weathering stages were classified as: 1) no cracking or flaking of the bones; 2) moderate flaking and small cracks on the cortical bone surface; 3) extreme flaking and large cracks penetrating the cortical bone. Breakage classification followed established criteria for long bone fragmentation [25]. The presence/absence of calcareous coating on the bone surface was recorded.

### Bioskeletal profile

Age-at-death and biological sex for each of the individuals was assessed. Age-at-death estimation followed multifactorial methods [26–35].

The cranial skeleton was too fragmentary for sex estimation. Fortunately, the three individuals identified preserved fragmentary remains of os coxae. The greater sciatic notch scores [36, 37] and the Phenice techniques [38] were used when possible to sex the individuals.

Stature estimation from bone length was limited, due to the fragmentation of the remains. When possible, stature calculations followed the equations developed for individual skeletal elements on Thai populations [39], and the regression formulae for skeletal height in modern Southeast Asians, including correction factors to estimate living stature [40, 41].

### Pathologies and trauma

Dental and postcranial lesions were identified and described following published criteria [42, 43]. To evaluate the cranial lesion previously described for TLB-1 [9], as well as dental pathologies, the samples were scanned at the National Laboratory for X-ray Micro Computed Tomography (CTLab) at the Australian National University (ANU) using a HeliScan MicroCT system with an optimized space-filling trajectory to yield sharp images at a resolution of 68–50 μm [44–46]. CT images were visualised and explored with Drishti v.2.7 software [47]. A virtual assessment of the cranial lesion was performed following criteria to assess the timing of the cranial trauma [48].

### Mortuary practices

Burial rites include three temporal acts: 1) preparatory practices before deposition; 2) deposition of the corpse, commonly in a burial container (e.g. jar, coffin), or a burial pit, positioning of the individual, and deposition of grave goods; 3) and post-burial actions such as grave disturbance, bone manipulation or removal, and reburial events (e.g. [1]). To reconstruct pre-burial actions, the burial context and presence/absence of defined grave outlines was recorded. Individual deposition was determined through the description of body positioning, skeletal articulation, grave associations, and burial type. Post-burial actions were evaluated based on skeletal element positioning and evidence of bone removal.

To describe body positioning, the general orientation of the complete skeletons, and of individual skeletal elements was recorded. The skeletal articulation assessment was based on the articulation stage of labile and persistent joints [1, 2, 5, 49].

Direct anthropogenic modifications of the deceased can leave marks on the bones in any of these temporal acts. The presence of pigment coating was recorded and photographed. Bone surface modifications presumably caused by anthropogenic actions (i.e. cutmarks or perforations) were visualised under high magnification microscopes (Dino-Lite AM4815ZT). High quality images were produced with an Olympus LEXT OLS5000 3D confocal measuring microscope, located at the School of Archaeology and Anthropology at the Australian National University. This microscope is equipped with two optical systems—colour imaging optics and laser confocal optics–that enable acquiring of colour and shape information, and high-definition images. It is equipped with a 405 nm short-wave laser diode light source and a high-sensitivity photomultiplier, with a shallow depth of focus enabling the measurement of sample surface irregularities. Linear profiles were obtained from high-resolution height and colour images at the mid-point (50%) of each modification, and analysed with the OLS50-BSW standard software.

For the identification of material associated with the grave, the presence of manuports (i.e. igneous rocks, beach cobbles, and ochre nodules) and grave goods was recorded, and illustrated in the burial diagrams.

## Results

A detailed description of the skeletal elements recovered in each of the burials from Tron Bon Lei is provided in the S2 File. To introduce these burials, we follow a chronological order, starting with the oldest burial (TLB-1) and ending with the most recent burial (E16b).

### Burial TLB-1

#### Bone preservation

The skeletal elements from burial TLB-1 appeared well preserved *in situ*. However, the excavation of the individual elements as well as the transport of the remains lead to fragmentation of most of the remains studied in the laboratory. The straight and irregular breakage morphologies, as well as the different colour of the breakage edge indicate the recent fragmentation of the bones. The cortical surfaces of the remains are covered in a brown calcareous coating.

#### Anatomical description

**Fig 3:**
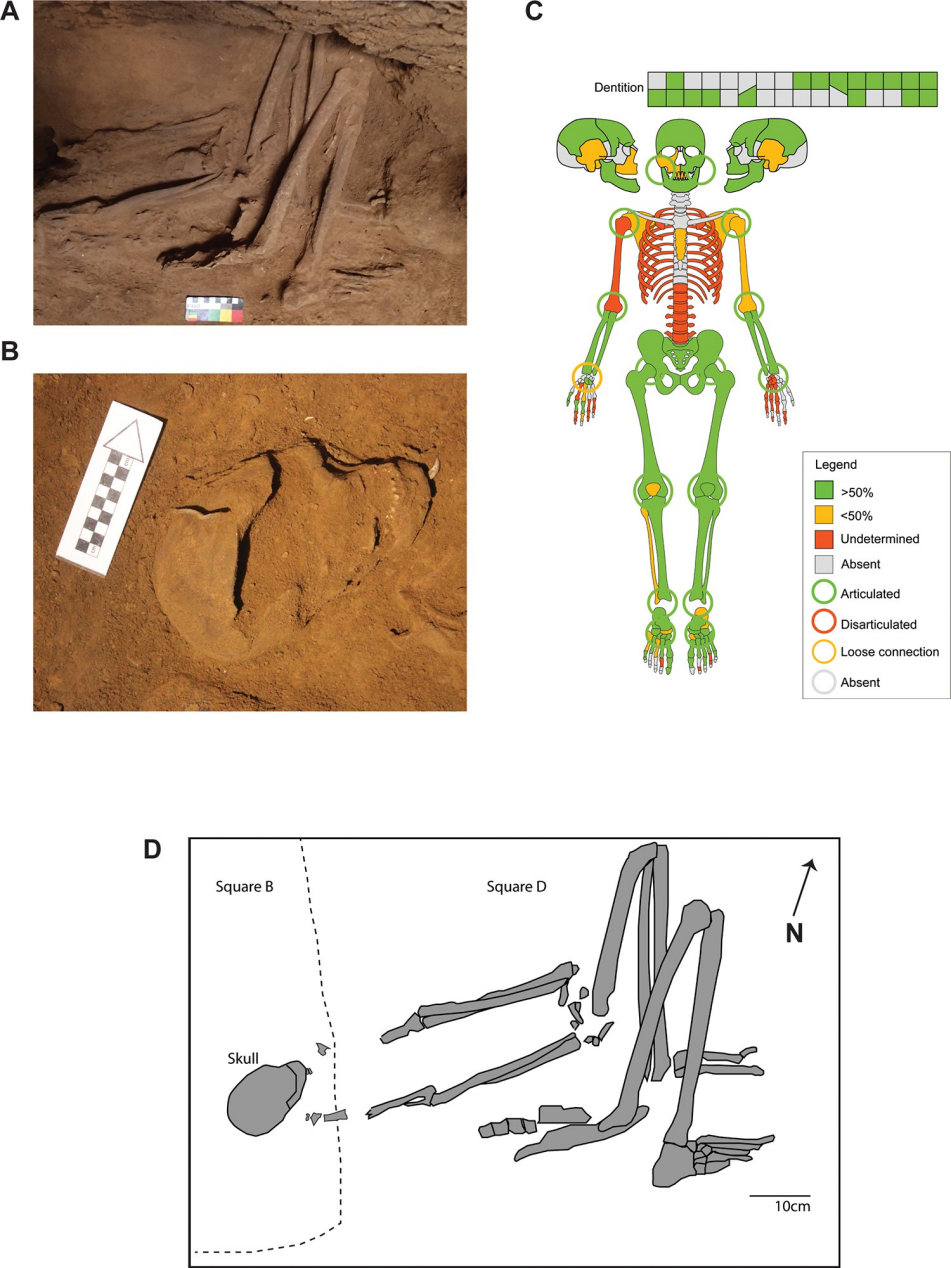
Skeletal elements from TLB-1. A) Complete articulated postcranial skeleton; B) Cranial remains recovered in 2014. Modified from Samper Carro et al. 2019 [9]; C) Skeletal diagram indicating the elements recovered (included those documented in 2014) and joint assessment (circles). D)Sketch drawing of the TLB-1 individual, including the elements excavated in 2014 (Square B, left) and the postcranial skeleton documented in 2018 (Square D, right). Dashed line indicates the original east limit of Square B in 2014.

The TLB-1 individual comprises an almost complete skeleton (Fig 3). The cranial remains, as well as both fragmentary scapulae, the cervical vertebrae and the diaphysis of the left humerus were recovered in 2014 [9]. The skeletal elements documented in 2018 consist of postcranial remains (S2 File).

#### Bioskeletal profile

Age and sex assessments were performed on the postcranial skeleton. Epiphyseal closure indicates an adult age for this individual. The auricular surfaces of the ilia present a rough and irregular surface topography, with a defined ridge delimiting the preauricular areas, placing this individual in Lovejoy’s phase 7 [30], with an estimated age of over 50 years old (Table 1). This age estimation coincides with the age assessment based on cranial suture closure, and dental eruption and attrition stages, previously published [9].

**Table 1:** Age-at-death Tron Bon Lei individuals.

	Burial TLB-1		Burial DE B2		Burial E16b	
Cranial	Phase/Score	Age range	Phase/Score	Age range	Phase/Score	Age range
Lambdoidal suture^a^	NA*	-	NA	-	0	<30.5
Dental wear^b^	I	45–55	I	45–55	D-E	20–30
Dental wear^c^	5+	>45	5++	>55	2/2+	17–25
Postcranial	Phase/Score	Age range	Phase/Score	Age range	Phase/Score	Age range
Pubic symphysis^d^	-	-	-	-	IV-VI	25–35
Auricular surface^e^	7	>50	-	-	2	25–29
Average age	minimum 45*		>55		20–25	

Results for age-at-death estimations based on cranial and postcranial ageing criteria.

^a^ [28]

^b^ [29]

^c^ [31]

^d^ [26]

^e^ [30]

* For a detailed description of cranial suture scoring for TLB-1, see [9]

The presence of a broad greater sciatic notch (score 2) [36] in both ossa coxae points towards a female, as was previously suggested based on mandibular and cranial traits [9]. The Phenice method was unsuitable to be used due to the high fragmentation of the right pubis, although a narrow symphyseal surface is observed.

Stature estimation based on the maximum length of the left tibiae yields a height of 151.9±5.94cm [39]. This estimation coincides with a living stature estimate calculated based on the tibia condyle-malleolar skeletal length of 151.4±3.38 [40, 41]. This height calculation places the TLB-1 individual within the stature range reported for the Liang Toge female skeleton, Javanese females, and modern Southeast Asian females [50, 51].

#### Body positioning

The skeletal remains show a general W-E orientation, with the head west facing north. The skeleton is lying on its left side. The upper limbs are symmetric, extended in front of the vertebral column, with the hands resting on the left os coxae, flexed inwards. The metacarpals and phalanges from both hands are commingled. The right os coxae was vertical, with the anterior side upwards, and the auricular surface facing north. The left os coxae was resting on its medial side, flat on the ground. Both legs were flexed, with the femora nearly parallel with the tibiae and fibulae. Both feet were in anatomical position, describing a 90 degree angle with the zygopodia.

#### Articulation assessment

The individual was interred in anatomical connection, with all joints articulated (Table 2). Both arms were rotated on the elbow, with the proximal segment of the ulnae resting on top of the proximal radius. The articulation of the left arm, including the distal humerus, complete radius and ulna, and carpals was preserved connected after excavation due to the presence of calcareous coating.

**Table 2:** Assessment of labile and persistent joints in the three individuals from Tron Bon Lei.

	Burial TLB-1			Burial DEB2			Burial E16b		
Joint name	Articulated	Disarticulated	Absent	Articulated	Disarticulated	Absent	Articulated	Disarticulated	Absent
Labile joints									
Temporo-mandibular	X				X				X
Hyoid			X			X			X
Cervical vertebrae (C3-C7)			X	X?				X	
Costo-sternal	X?					X		X?	
Scapulo-thoracic	X?			X?					X
Hands (carpals, metacarpals, phalanges)	X?			X				X	
Patellae	X			X					X
Feet (tarsal, metatarsals, phalanges)	X			X			X		
Persistent joints									
Atlanto-occipital	X?			X?					X
Humero-ulna	X			X*					X
Thoracic vertebrae	X?			X?			X?		
Lumbar vertebrae	X?			X			X		
Lumbosacral	X?					X	X		
Sacro-iliac	X					X	X		
Acetabulo-femoral	X			X					X
Tibiofemoral	X			X					X
Talocural	X			X*				X	
Talocalcaneal	X			X*			X		

Classification adapted from [1, 5]

? = uncertain

* = based on one side.

#### Anthropogenic modifications

No anthropogenic modifications were recorded.

#### Pathologies and trauma

**Fig 4:**
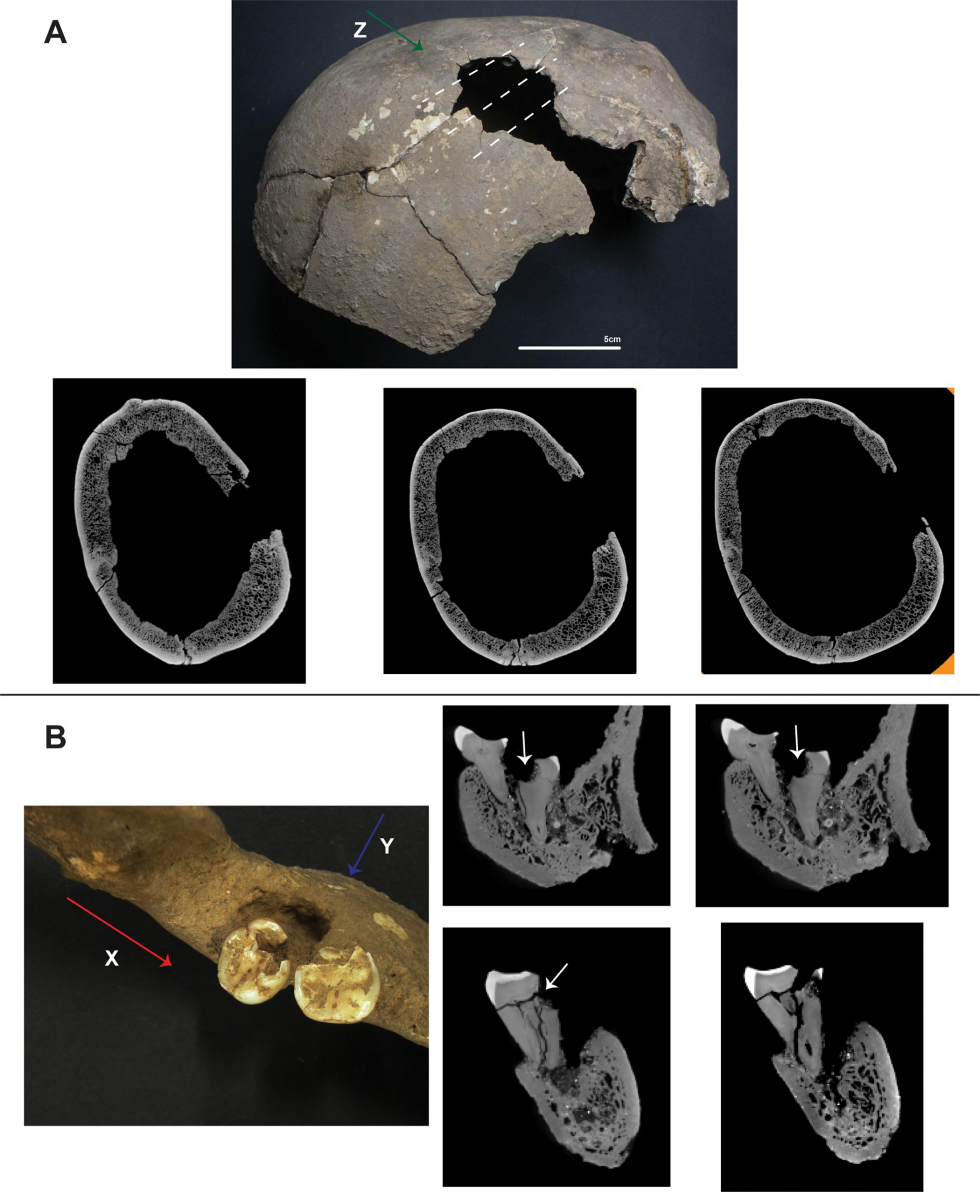
Micro CT-scans from TLB-1. A) Photograph of lesion on the right parietal (top) and CT images showing top (bottom left), middle (bottom centre) and bottom (bottom right) morphology of the lesion on the Z plane. Dashed lines indicate the location of the scanned images; B) Detail of dental lesion and CT images on the X (top) and Y (bottom) planes showing the morphology of the lesion (white arrows).

A cranial lesion was previously identified on the right parietal, above the hat rim line [9] (Fig 4A). Macroscopically, the lesion displays slight peripheral out-bending, with some small radiating fractures and concentric fractures, and loss of the inner cranial table, which could correlate with a trauma from outside the cranial vault. It presents rough and irregular edges, covered with calcareous coatings. The lesion largest dimensions are 3.5cm (posterior-anterior) and 3.4cm (superior-inferior).

The virtual assessment of the micro-CT images of the cranium from TLB-1 indicates that this lesion was produced post-mortem (Fig 4A). The preponderant texture is rough, with an irregular preponderant outline. Edge morphology displayed right angles, with the fracture angle unable to be assessed due to post-depositional fragmentation. There is no presence of relationship to path of least resistance, no signs of plastic response and absence of hinging. All these traits coincide with those commonly observed on post-mortem fractures [43].

Dental pathologies included advanced wear on upper and lower dentition (including anterior and molar teeth), antemortem tooth loss, and gross carious lesion on the mesobuccal side of left lower third molar, forming a periapical cyst (Fig 4B). Although the site of caries initiation cannot be confidently identified, CT imaging reveals that the large cavity progressed involving the dentine and the pulp chamber. The infection travelled through the root canal, causing the periapical cyst, displacing the third molar (Fig 4B).

**Fig 5:**
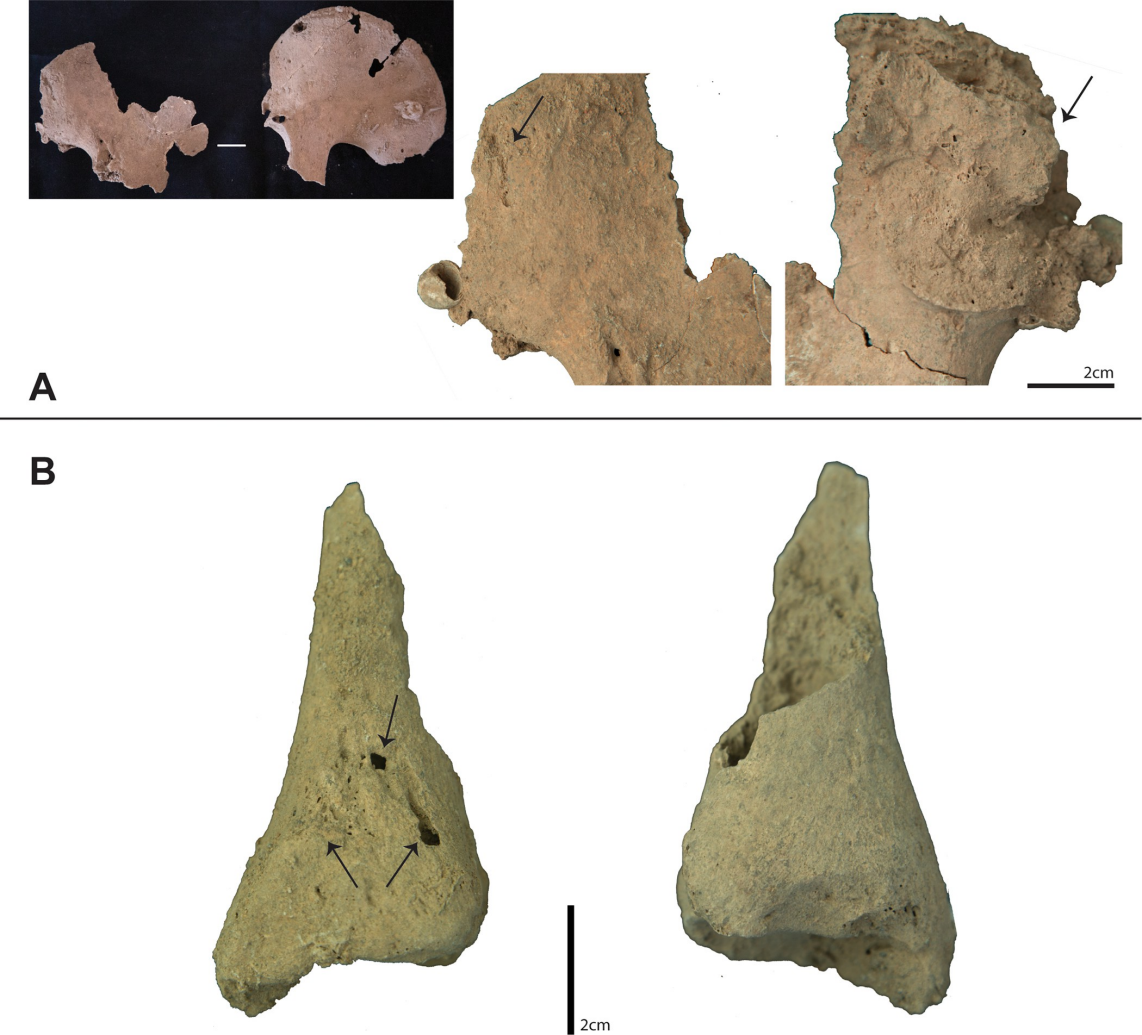
Postcranial pathologies in TLB-1. A) Lesion in right ilium. Comparison between right and left ilia (rectangle). Note the abrupt end of the right ilium after the greater sciatic notch. Detail of the cloacae (left, black arrow) on the lateral, and new bone formation (right, black arrow) on the auricular surface on medial; B) Detail of right distal tibia with two cloacae and pitting on the posterior side. Note spiral breakage morphology.

In the postcranial skeleton, periosteal new bone formation was recorded in the right os coxae and the right tibia. The right iliac tuberosity displays a rough surface formed by new bone deposition, with two cloacae identified on the lateral side (Fig 5A). Even though calcareous coating hampers the detailed observation of the posterior border of the ilium, it appears than this lesion has resulted in a reduction of the ilium size, which finishes on the preauricular sulcus, with the posterior iliac spine absent.

Periosteal pathologies were recorded on the anterior cortical bone of the distal tibia, comprising thinning of the cortical, slight swelling of the distal shaft, and two cloacae (Fig 5B). This portion displays a spiral breakage outline, which indicates the fracture was produced on fresh bone. The incomplete conjoining with the shaft, with fragments of cortical bone missing, as well as the lack of bone formation evidence suggest that this fracture was produced soon after the death of the individual, presumably as a result of soil pressure on a weak portion of the bone affected by the described lesions, which caused cortical thinning.

#### Grave associations

**Fig 6:**
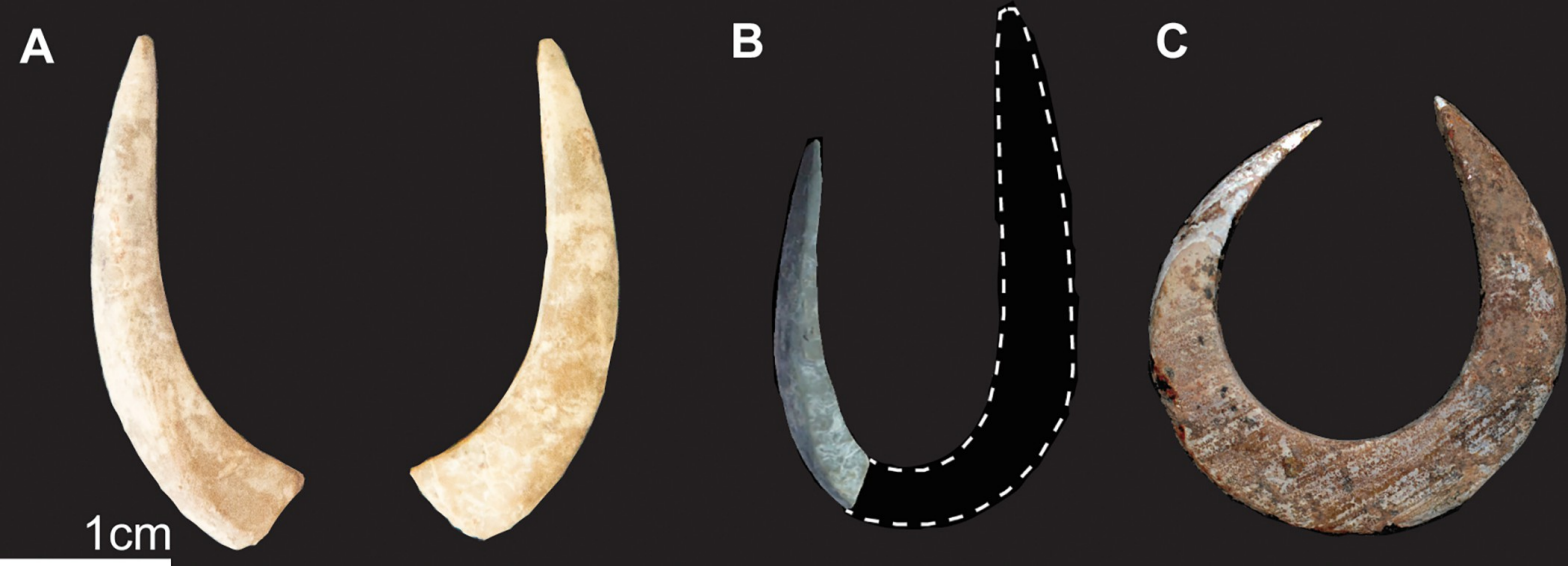
Fish-hooks from TLB-1. A) Broken fish-hook shaft recovered during the 2018 excavations; B) Example of fragmented jabbing hook (J-shaped) documented in 2014 from the TLB-1 burial; C) Example of rotating hook documented in 2014 on the neck of TLB-1. B and C modified from O’Connor et al. 2017 [8].

Five shell fish-hooks and a perforated bivalve were recovered around the neck of the TLB-1 individual [8]. An additional broken fish-hook was documented in 2018, identified as a broken J-shaped hook (Fig 6).

Cobbles with ochre coating were recorded above and beside the cranial remains [9], with no additional cobbles documented around the postcranial skeleton.

#### Interpretation of burial type

The individual from TLB-1 was interred in a primary flexed burial, lying on its left side and with no evidence of post-mortem manipulation of the skeleton other than the inclusion of grave goods (i.e. fish-hooks) and manuports (cobbles with ochre coating).

### Burial DE B2

#### Bone preservation

The skeletal elements recovered from burial DE B2 are poorly preserved, covered in calcareous coating with large cracks penetrating the cortical bone. The remains present post-depositional fragmentation patterns, identified as orthogonal and columnar breakage morphologies in the long bones, as well as different coloration of the breakage edge. This indicates that fragmentation occurred long after bone deposition, either during excavation of the remains or due to transport.

Several long bones present collapsed diaphyseal surfaces and deep cracks through to the medullary cavity, suggesting post-depositional crushing due to soil pressure and/or the weight of the rocks placed above the skeleton.

#### Anatomical description

**Fig 7:**
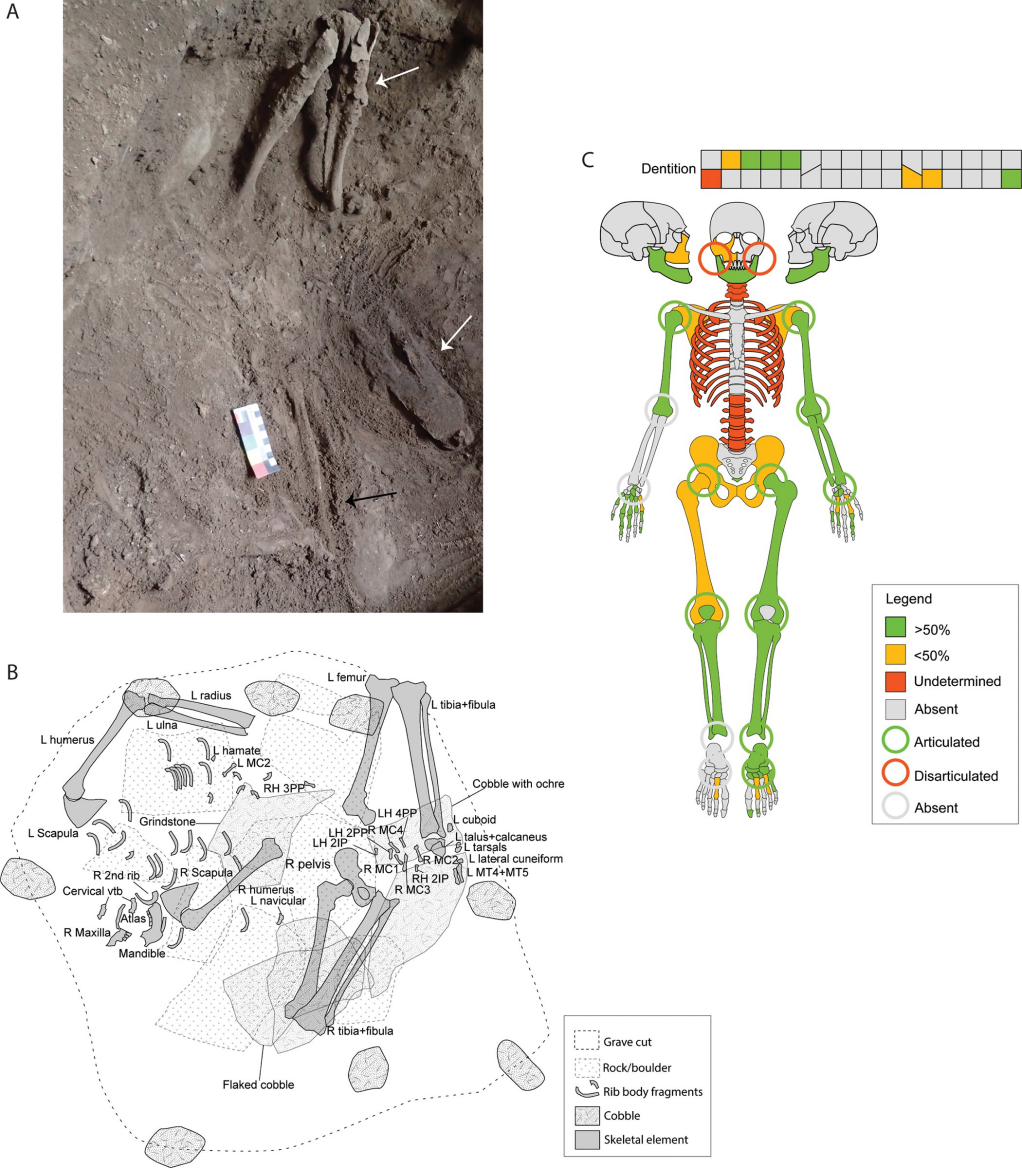
Skeletal elements from DE B2. A) General view of burial showing vertical legs (white arrows) and articulated upper right limb (black arrow); B) Sketch drawing of the position of the skeletal elements based on the spatial plotting of elements during fieldwork. Only those elements plotted are illustrated. Dashed lines in rock/boulders indicate that these elements were on top of the skeletal remains, removed before excavation of the burial; R = Right; L = Left; RH = Right hand; LH Left hand; MC = Metacarpal; MT = Metatarsal; PP = Proximal phalanx; IP = Intermediate phalanx; C) Skeletal diagram indicating the elements documented in DE B2 and joint assessment (circles).

The individual from DE B2 consists of a partially complete skeleton, including a partial right maxilla and fragmented mandible, and an incomplete postcranial skeleton (Fig 7; S2 File).

#### Bioskeletal profile

The portion of long bones preserving fused epiphysis fragments indicates the adult age of this individual, confirmed by the presence of erupted lower third molars.

Regarding dental wear patterns, the upper and lower dental elements identified present an extreme attrition. The right upper second premolar is extremely worn, with a complete loss of the crown. The right first upper molar has complete occlusal dentine exposure. The enamel of the right second upper molar is only preserved in the paracone, with dentine exposure on the metacone, and broken protocone and hypocone. The left upper first premolar and molars have their dentine exposed, with the enamel only preserved on the buccal and distal sides of the first premolar.

The left lower first and second premolar have exposed dentine. The lower third molars have dentine exposure, with an unusual wear pattern where a small portion of the lingual and labial enamel is preserved.

These tooth wear patterns would exceed those describe in Lovejoy’s phase I [29], with the third molars patterns matching the wear scores 5++ [31]. These results indicate an estimate an age at death of over 55 years old (Table 1).

The left coxae presents a broad greater sciatic notch, scoring 2 [37]. This likely indicates a female, although this assessment should be considered cautiously due to the absence of primary sexual dimorphism indicators.

Despite the presence of long bones, their fragmentation, with none of the bones preserving its complete length and circumference, precluded estimation of stature for this individual.

#### Pathologies and trauma

**Fig 8:**
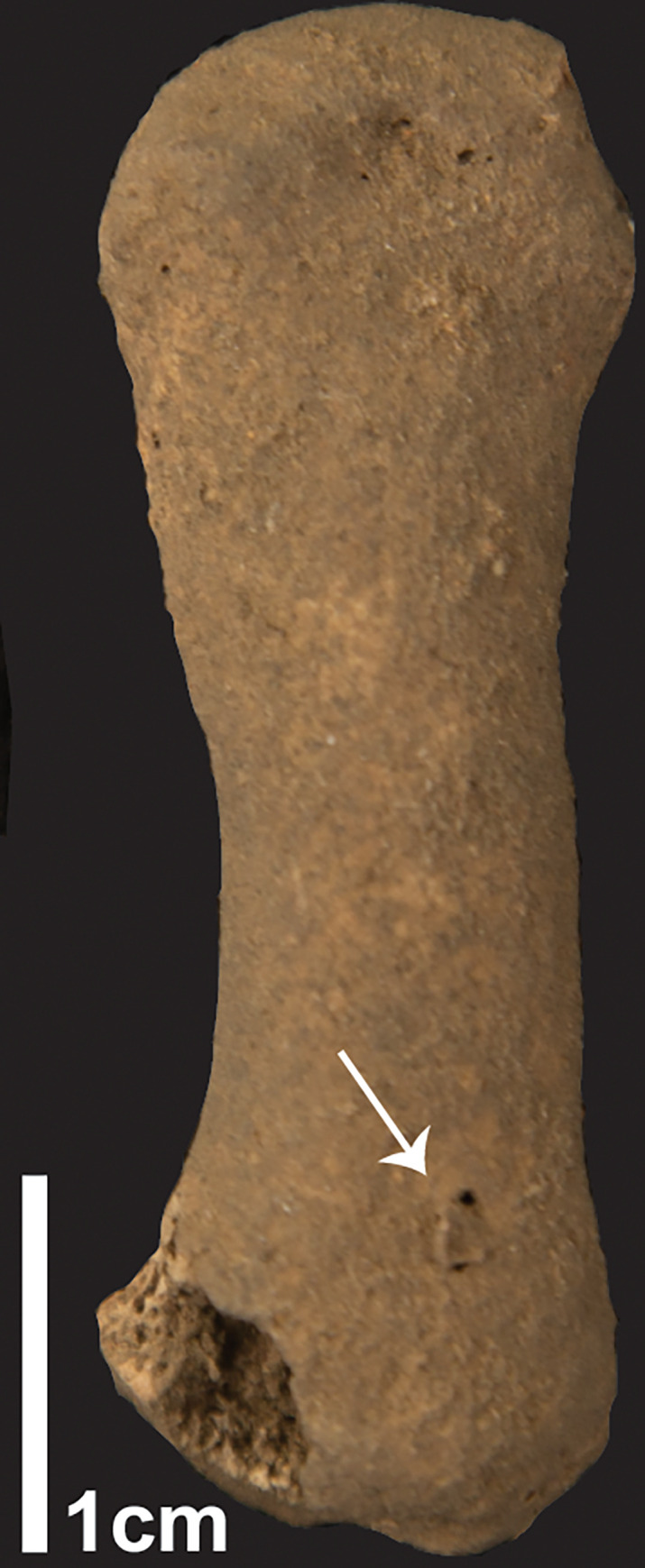
Right first metacarpal from DE B2. Perforation and unidentified intrusive object (white arrow).

Two anomalies were identified in the DE B2 individual. The right upper permanent first premolar is unerupted with a completed root formation. The right first metacarpal presents a small perforation on its dorsal surface, with an unidentified intrusive object stuck in the perforation (Fig 8). Without the extraction of the object, and consequently damage to the bone, a more detailed description of the intrusive object is not available at the moment. Currently, we consider unnecessary the extraction of the object for further investigation. Nevertheless, observation under high magnification suggest that it corresponds with a hard object of unknown material. The general morphology of the metacarpal is unusual, with an uneven shape of the shaft.

#### Body positioning

The DE B2 burial had a general W-E direction, with the cranial elements (i.e. maxilla and mandible) at the west of the burial. The upper body is in supine position laying roughly at the same level as the os coxae, with the scapulae, upper limbs, cervical vertebrae, and ribs in anatomical connection. The upper limbs were symmetric, extended parallel to the torso. Both legs were on the east side of the burial, flexed with the knees up, and the left femur and tibia oriented vertically. The right leg was vertical and splayed outwards, with the knee at the edge of the grave cut. The left tarsal series appeared connected to the zygopodia.

#### Articulation assessment

The skeleton is partially articulated (Table 2). The temporo-mandibular joint was assessed as disarticulated due to the absence of fragments from the temporal bones identified. The right and left shoulders were recorded as articulated during excavation, as well as the left elbow and wrist. The distal portion of the cervical vertebrae (C-3 to C-7) and most of the ribs from the right side appeared in anatomical connection *in situ*. The femora were articulated with the acetabulum, with both legs articulated at the knee joint. The left tarsal series was articulated with the distal tibia, with the calcaneus and talus still connected due to the presence of calcareous coating.

#### Anthropogenic modifications

No clear anthropogenic modifications were identified, although one of the right rib bodies has a light red pigmentation that could be ochre staining.

#### Grave associations

**Fig 9:**
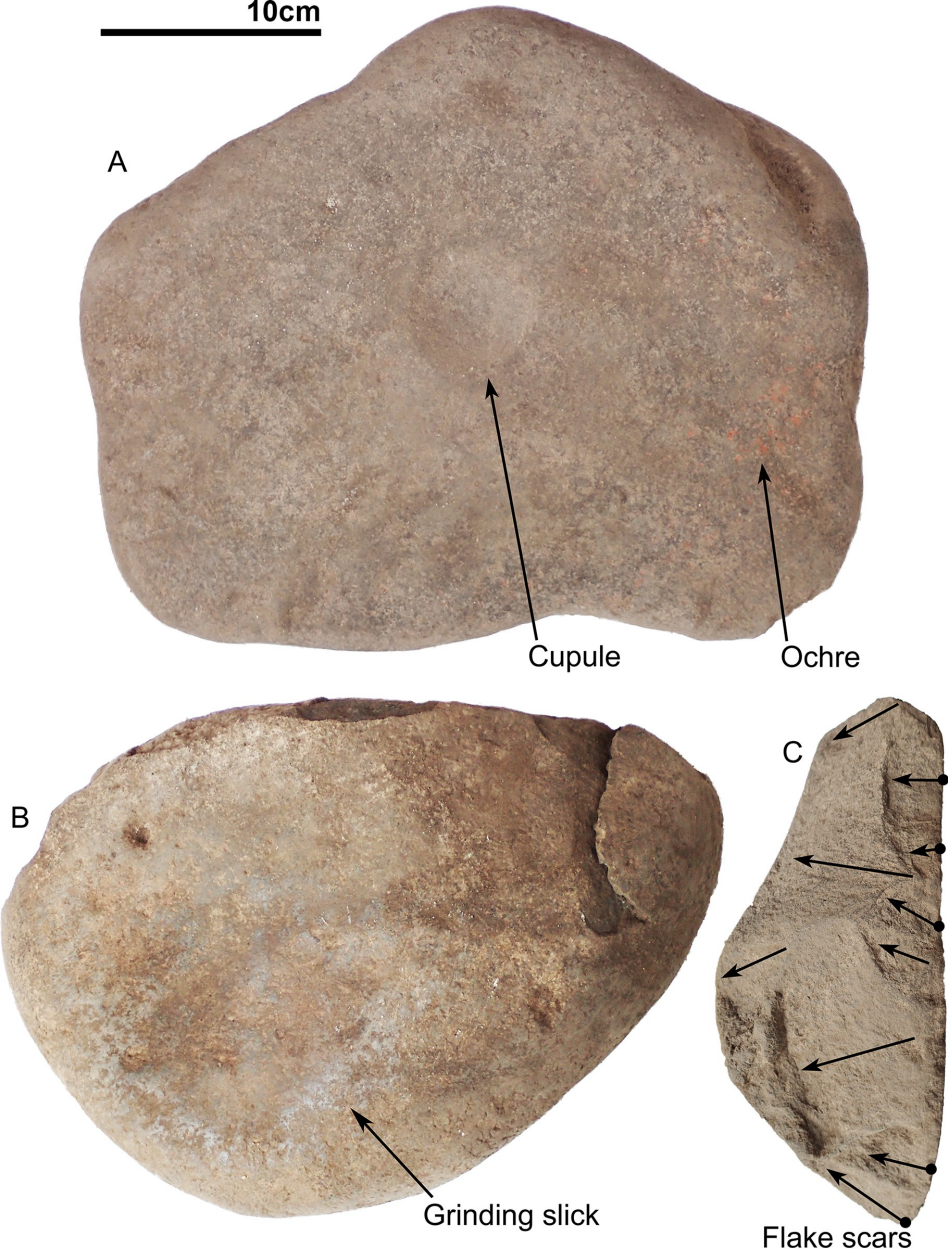
Cobbles from Tron Bon Lei burials. A) Ochred cobble with a regular cupule in the centre, that was placed directly on top of burial E16b; B) Grindstone associated with burial DE B2; C) Flaked surface of a cobble associated with burial DE B2. See Fig 7 for location of these cobbles in DE B2.

Several large rocks were placed above the grave cut. One cobble was recorded on the top part of the burial, with three other cobbles recovered from the grave infill. Several rocks, tightly wedged together as well as an additional cobble were documented at the base of the burial. One of the cobbles, with ochre coating, has been flaked (Fig 9), while another was broken in half. Another large cobble seems to be a grindstone with a polished and lightly dished area 17cm in diameter on one of its sides (Fig 9). Basalt and chert flakes were documented throughout the burial infill, with two small pieces of ochre recorded.

#### Interpretation of burial type

The DE B2 is interpreted as a primary burial, based on the body position, and anatomical connection of the different skeletal segments recorded. The limited presence of cranial elements could be the result of post-depositional damage and fragmentation of these elements, with some of the densest portions (mainly dentition, mandible and fragmentary right maxilla) being preserved. The rest of the cranial skeleton could be present in the form of small fragments of flat bone recorded in the laboratory, but poor preservation hampered assignment of these further.

The position of the lower limbs suggest that the individual was interred in a seated or squatted position, presumably maintained throughout the decomposition process through either the use of wrapping material, an organic burial container, or rocks as support. The supine position of the upper body has two possible interpretations. The upper body was separated from the lower body during interment, which we consider unlikely due to the presence of articulated joints. A second option is that the upper body collapsed after decomposition of the soft tissue, due to the lack of enough support on the back and delayed sediment deposition inside the grave cut. Additionally, the collapse of the upper body, as well as the presence of a cobble on the top part of the burial and large rocks above the grave cut could have contributed to the destruction of the cranial elements.

### Burial E16b

#### Bone preservation

The skeletal elements from burial E16b are poorly preserved. The cortical surface of the bones is flaky and fragile, which hampered cleaning, as even dry brushing of the adhered sediment resulted in further fragmentation. The cranial vault was crushed post-depositionally, comprising multiple fragments. Most of the fragments have lost the inner and/or outer cranial tables, with the diploid bone exposed. This made the reconstruction of the cranium complicated, although some portions were identified. The fragility of the remains is demonstrated by the loss and high fragmentation of the roots of the upper dentition. The absence of long bones impeded the assessment of breakage patterns.

#### Anatomical description

**Fig 10:**
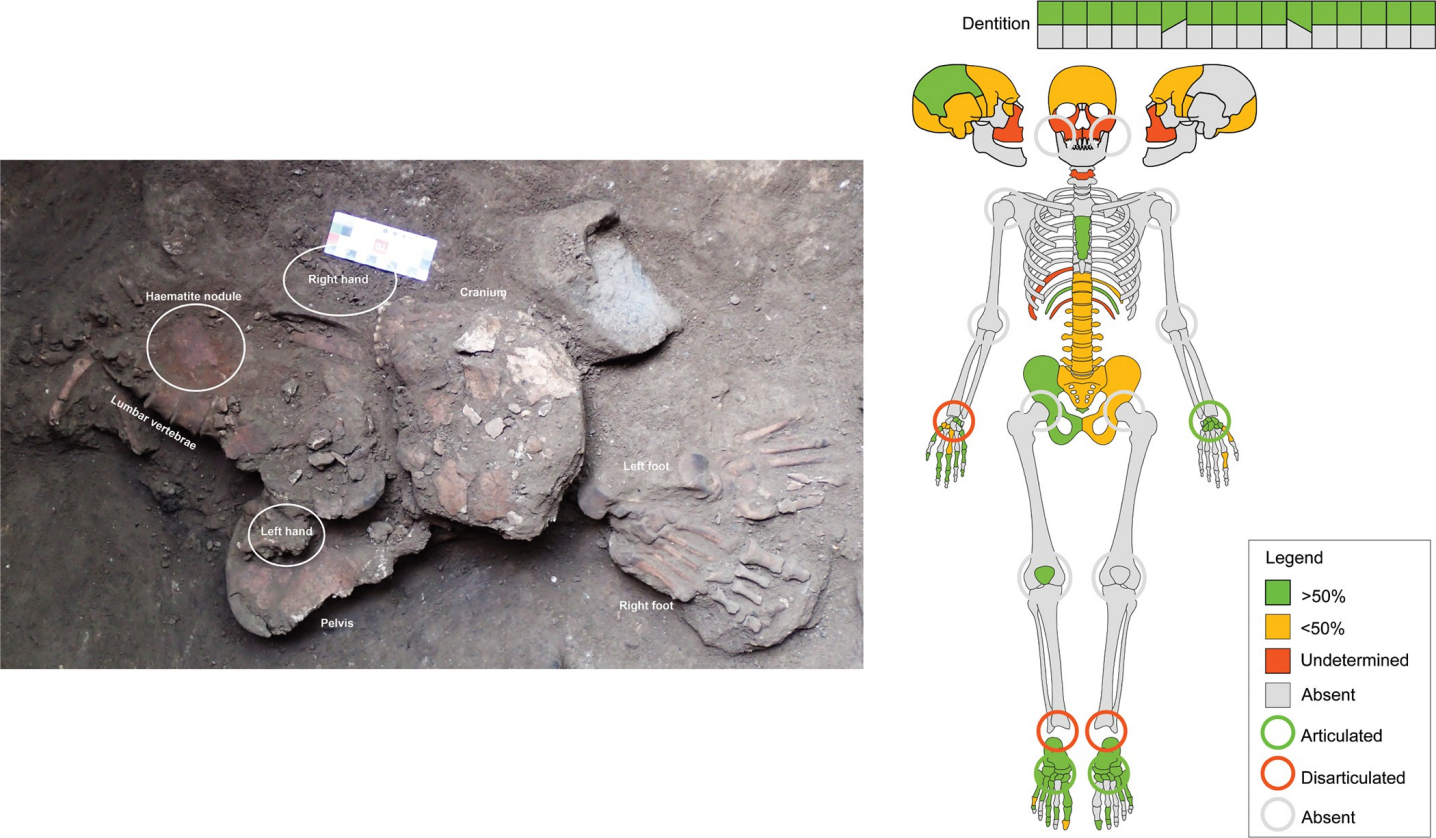
Skeletal elements from E16b. Burial E16b *in situ* (left). Skeletal diagram indicating the elements documented in the burial and joint assessment (circles).

The individual from E16b consists of an incomplete skeleton (Fig 10), including a fragmentary skull, axial skeleton and distal limb elements (i.e. autopodia). No mandible or long bones were recovered (S2 File)

#### Bioskeletal profile

Despite the high fragmentation of the cranium, the preservation of conjoining portions of the lambdoidal suture indicates that this suture was not closed at the time of death [28]. The rest of sutures were unable to be assessed.

The molar dental wear patterns observed correspond to stage D and E, which estimate an age at death ranging from 20 to 30 years old [29], or 17 to 25 years old [31]. The developmental state and slight wear in the upper third molars correspond to the age at death of this individual over 20 years old.

Based on phases of pubic symphysis modification [26], the presence of a developing border as well as the limited billowing observed place this individual between phases IV, V and VI (25–35 years old), although since only a small portion of the left pubis was preserved this age estimation should be considered with caution. In the right pelvis, the auricular surface yields a reduction of billowing but a reduced granularity, placing the individual in phase 2 (25–29 years old) [29].

The combination of cranial and postcranial methods to age the E16b individual resulted in an estimated age of death of 20–25 years old, corresponding to a young adult (Table 1).

The fragmentary right pelvis was used to estimate the biological sex of this individual. A narrow greater sciatic notch gives this individual a score 4/5 [36, 37], which would correspond to a male. This observation was compared to the results obtained for the left portion of the pelvis through the Phenice technique [38]. The lack of subpubic concavity, as well as a wide ischiopubic ramus, support the male assessment.

#### Pathologies and trauma

**Fig 11:**
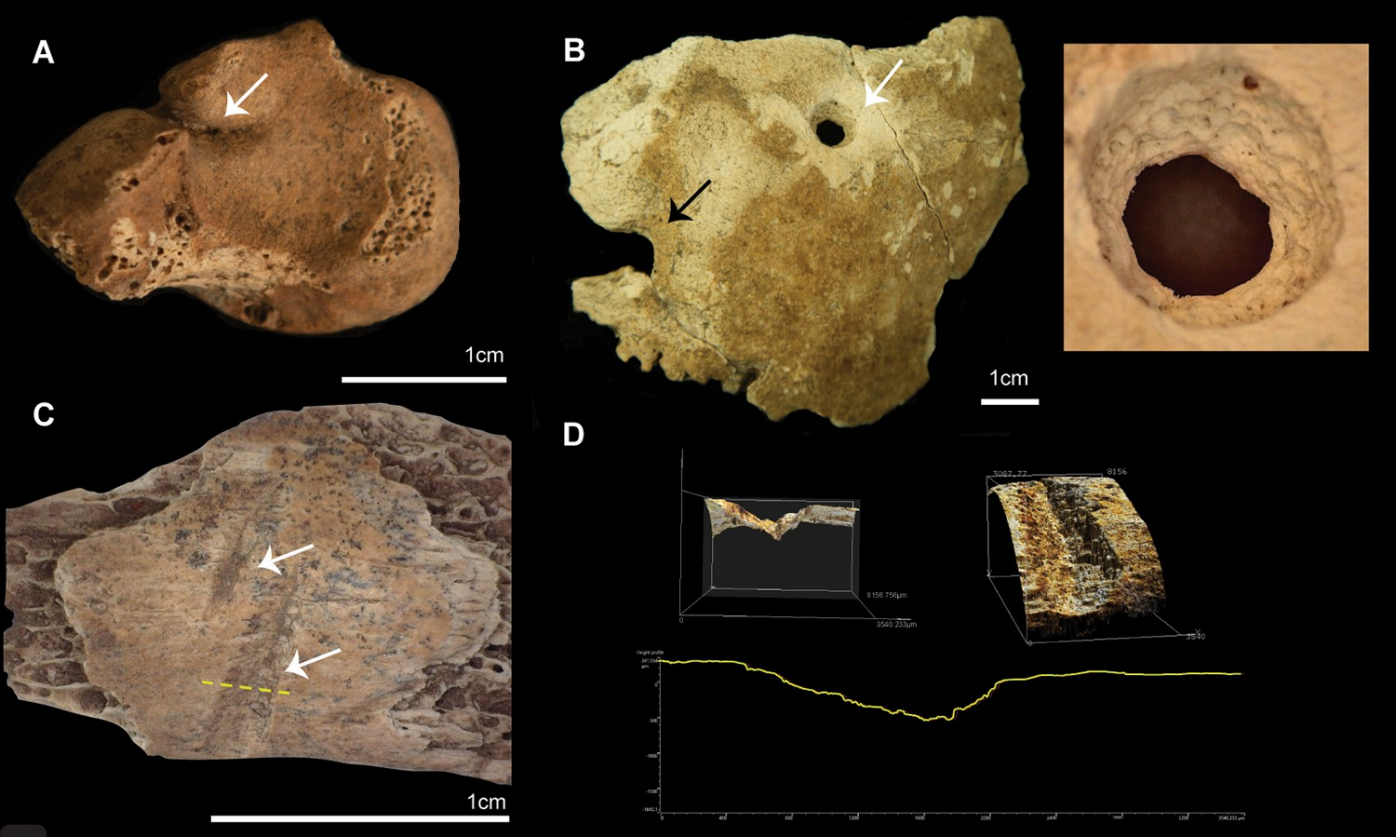
Pathologies and modifications in E16b. A) Left scaphoid with evidence of healed fracture; B) Left, fragment of right parietal with circular perforation and (white arrow) and incomplete outline of a second perforation (black arrow). Right, detail of circular perforation; C) Rib fragment with cutmarks. Yellow dotted line indicates the exact position of the profile represented in D. D) 3D Confocal images at 10x of colour data (top) and (bottom) profile of the cutmark analysed (yellow dotted line and white arrow in C).

The left scaphoid presents a healed fracture (Fig 11A). A circular perforation was identified on the right parietal, near the parietal tuber, and with the partial external diameter of a second perforation identified near the first perforation (Fig 11B). The complete perforation presents a conical shape, with a larger diameter on the outer cranial table than in the inner table. This shape suggest that the perforation originated from outside the endocranium. No clear striae were identified inside the perforation. The direction of the perforation (outside-inside), as described by its conical shape, as well as the presence of a second perforation nearby, suggest that these modifications could have been anthropogenic, as no evidence of insects’ boring activity was identified in this cranial fragment, or any other skeletal element. Nevertheless, the erosion of the internal walls impedes the identification of striae to assess intentional drilling (Fig 11B).

#### Body positioning

The E16b individual presented a general E-W orientation, with the head in the centre of the grave cut. The position of the body is difficult to determine due to the absence of long bones. The preserved skeletal elements appear relatively commingled, with the cranium facing north placed over the pelvic girdle, the preserved vertebral column towards the west, and feet east, facing away from the cranium (Fig 10). The elements from the right hand were placed north of the vertebral column, close to the maxilla, with the incomplete left hand placed over the right pelvis.

#### Articulation assessment

Different skeletal segments were recorded articulated (Table 2). The thoracic and lumbar vertebrae, the sacrum and coccyx, and right pelvis were in anatomical connection. The feet were articulated, including the calcanei and tali. The left carpal series was articulated based on the geospatial distribution of the remains. The geospatial distribution of the right carpals and phalanges suggest that they were disarticulated at the time of excavation, although their articulation state when buried cannot be assessed (Fig 10).

#### Anthropogenic modifications

Several fragmented ribs and vertebral bodies displayed ochre staining. A fragment of a rib body presents two linear marks identified as cutmarks (Fig 11C). This modification yields an asymmetrical cross-section with step-like striations (Fig 11D). A reddish residue was observed on the upper dentition, more prominent on the buccal side of the incisors and molars. Preliminary observations under a high magnification microscope, and analysis through portable X-ray fluorescence, anticipate that this residue does not correspond with the chemical signature of ochre staining, as no iron was detected. Based on the reddish colour and position (buccal side) of the residue, we hypothesise that it might correspond with betel nut (Areca nut) chewing and consumption, or deliberate application of the residue prior or post-mortem. The residue was scraped from the teeth, avoiding damage of the enamel, and it is currently being analysed through a gas chromatography mass spectrometer (GC MS) to determine whether it is organic (betel nut) or inorganic (ochre).

#### Grave associations

Eight large angular rocks and rounded cobbles were associated with the burial, placed around the edge, on top of the burial cut or directly associated with the skeleton. All eight rocks have ochre on them, with four of them featuring cupules ~6cm diameter and ~0.5cm deep. A large cobble with a cupule and ochre coating (Fig 9) was placed directly on top of the cranium, which likely caused its fragmentation. Another cobble with red ochre coating was recorded on top of the grave cut and removed prior to excavation of the skeleton. A large ochre nodule placed on top of the thorax was found during excavation (Fig 10).

#### Interpretation burial type

The absence of long bones and mandible, as well as the preservation of articulated skeletal segments (i.e. feet and lower section of the vertebral column) indicated that this interment could be interpreted as a delayed primary burial, where the skeleton was exposed prior to final deposition, or a secondary burial, where bones were re-buried after removal of elements previously buried in a different location.

The poor preservation state of the skeletal elements, with a flaky appearance, could be related with sub-aerial exposure of the remains prior to burial, or otherwise, it could be result of post-depositional decay of the bones.

The presence of clearly identifiable cutmarks in one of the rib bodies suggests intentional dismembering of the body before burial. The decomposition stage before burial is uncertain, although the preservation of connected vertebral segments and feet bones suggest that some soft tissue was likely preserved at the time of interment.

## Discussion

The application of archaeothanatology to the study of human burials brings together information from the excavation with the data recovered from the laboratory analysis. In Southeast Asia, there is an increasing number of detailed studies on burial practices, with researchers discussing aspects such as body positioning, skeletal articulation, palaeohealth, and grave associations, in addition to traditional reporting of bioskeletal profile data [52–58]. This emerging interest in the study of burial contexts allows the new burials from Tron Bon Lei to be contextualized in comparison with other chronologically- and geographically-close mortuary manifestations. Nevertheless, singular aspects of the individuals interred in Tron Bon Lei deserve to be discussed.

### Burial practices at Tron Bon Lei

The three new burials excavated in squares D and E from Tron Bon Lei allow us to discuss shifts and continuities in social behaviour in the Lesser Sunda Islands from the Late Pleistocene. The vertical and horizontal distribution of the three burials, separated by c.70cm in depth and no more than 30cm between the horizontal spatial distribution of the second and third burial, indicate a recurrent use of the rockshelter as a burial context from, at least, the terminal Pleistocene to the mid-Holocene. This is supported by the chronostratigraphy of the burials, dating to ca. 12kya cal BP (TLB-1); ca. 10kya cal BP (DE B2) and ca. 7.5kya cal BP (E16b).

Even though the three burials share a similar location in the rockshelter, the differences between burial types, body positioning, and associated grave material suggest shifts in socio-cultural practices from the Pleistocene to the mid-Holocene.

The oldest individual excavated in Tron Bon Lei (TLB-1) comprised a primary flexed burial, with no evidence of post-depositional disturbance. Nevertheless, this individual denotes an intensive treatment of the skeletal remains before burial, being the only individual identified with items of personal equipment placed on the body. The meaning behind the technological attributes of these fish-hooks has been discussed elsewhere [8]. However, some of the physical characteristics of this individual contribute to previous interpretations which were based solely on craniometrics [9].

The recovery of the postcranial skeleton from TLB-1 permits us to confirm the female sex of this individual, which is relevant to discussions about gender roles in prehistory [8]. Additionally, the stature estimation places this individual within the range of Javanese females and the Liang Toge individual, being considerably shorter (c.15cm) than the female from Liang Lembudu [45, 59]. These calculations confirm the small size of this individual as defined by the craniometrics. As previously suggested, small dimensions could reflect the presence of a somewhat genetically isolated population on these islands [9].

Recent genetic research indicates complex patterns of admixture in the Lesser Sunda Islands, with evidence of genetic heterogeneity and survival of ancestral genomic components in modern populations [59–61]. Nevertheless, based on phylogeographic analyses of mitogenomes, a demographic change is identified around c. 15kya, indicating westward population movement from New Guinea in the east, and coinciding with rising sea levels after the Last Glacial Maximum [59]. In addition, phylogenetic analysis of aDNA from a mid-Holocene burial on Sulawesi indicates eastward genetic movement from mainland Asia across Wallace’s Line, some time prior to 7.5kya [62]. These results, coupled with evidence for maritime obsidian transport [18], and shared inter-island bead and fish-hook traditions in the terminal Pleistocene and earlier Holocene [63], indicate high levels of connectivity and movement during the occupation of Tron Bon Lei. Cultural transmission may also be indicated by the variations in mortuary practices observed at Tron Bon Lei across the 5,000 year period spanned by the burials described here.

Genetic and proteomic sampling of the individuals presented in this paper, and two additional individuals previously published (TLC-1 and TLC-2), is currently underway. If successful, the results from these analyses might contribute to the interpretation of the causes for the small dimensions of the individuals from Alor and neighbouring Wallacean islands. Additionally, these results could provide evidence for different migrations that contributed to the genetic admixture observed in modern Alor populations.

The dental pathologies identified in TLB-1 inform on subsistence practices during the terminal Pleistocene. Severe dental wear is a common trait in hunter-gatherer communities, with Pleistocene individuals generally displaying steeper wear facets and oblique wear angles [62, 64]. These wear patterns have been related to environmental abrasiveness and food processing methods, such as desiccation that requires long chewing time, as well as ingestion of exogenous materials, such as sand and grit [62].

Ante-mortem tooth loss can be caused by several factors, including trauma or metabolic diseases, but in most cases is due to periodontal diseases [63]. The gross caries identified in the lower left third molar, affecting the root canal and resulting in a periapical cyst, might have contributed to tooth loss, especially due to the proximity between the lesion and the lost first molar. Additionally, the consumption of cariogenic wild plant foods, such as nuts, might lead to severe consequences on oral health [65]. The inclusion of plant foods in the diet of the TLB-1 individual is supported by the published isotopic values, while the consumption of abrasive particles in consumed marine resources and its relationship with dental lesions has been suggested [22, 66]. Future analysis of dental macro and microwear from the three individuals reported here, will contribute to characterising the diet of the Alor populations from the terminal Pleistocene to the mid-Holocene.

The position of the lower limbs in the second burial (DE B2) has been interpreted as evidence of a seated or squatting position. A thorough archaeothanatological study of over 200 seated burials from southern Peru provides criteria to evaluate the processes affecting the body position of the DE B2 individual [67]. The high fragmentation and virtual absence of the cranium coincide with observations on common disassociation and post-depositional natural movement of this element in seated interments. In the Peruvian burials, crania appeared disassociated and moved from their anatomical position in over 35% of the burials, being absent in 28% of the interments [67]. In these burials, post-depositional movement of the crania is explained as a result of the crania being the least stable element, due to their weight and high position in seated burials, as well as anthropogenic removal of crania with mandibles in partially skeletonized remains. In DE B2, the identification of a partial right maxilla, as well as the mandible dissociated and moved from its anatomical position conform with the first scenario.

The position of the arms, extended along the body, might be the consequence of voids in the grave fill or container, resulting in the movement of the arms by gravity while the joints are still connected [67]. The displacement of the upper body is not unusual in seated burials, as this segment is susceptible to movement by gravity when the decomposition of the soft tissue outpaces the rate of sediment deposition [67]. We consider that the presence of large rocks above the skeleton will have contributed to the collapse of the upper thorax and the damage and fragmentation of the skull, as indicated by the collapsed cortical surfaces observed on the long bones and the fragmentation of the mandible. The post-depositional movements described suggest that the individual was buried wrapped or inside an organic burial container, which decomposed prior to soft tissue decomposition, or otherwise, that the wrapping material did not apply enough pressure on the decomposing body to counteract gravity.

While no clear evidence of ochre staining was observed on the skeletal elements, the presence of ochre coated rocks and cobbles suggest that ochre processing was part of the burial rites. The presence of a large cobble with a concave polished surface without ochre staining might be related to plant processing.

The top burial (E16b), has been interpreted as a delayed primary burial or a secondary burial. The poor preservation state of the skeletal elements, brittle and flaky, could be interpreted as potential evidence for sub-aerial exposure of the skeletonized remains. Nevertheless, the preservation of articulated labile joints in the feet, suggests the presence of connective tissue when these elements were buried, which would not conform with an extended exposure of the complete skeleton. Additionally, the identification of cutmarks in one of the rib bodies suggests some dismembering of the skeletal segments. Based on these observations, we postulate that pre-burial treatment of the individual from E16b might have included the dismembering and removal of skeletal elements before complete decomposition of the soft tissue. Nevertheless, cutmarks were only confidently identified in a single element–the rib body illustrated in Fig 11. As such, dismembering activities could have been sporadic, or the absence of cutmarks could be just a consequence of the poor preservation of the cortical bone in most elements, hampering the identification of bone surface modifications. The presence of large rocks intentionally placed on top of the burial cut suggest that the removal of skeletal elements is not related to post-depositional disturbance of the burial.

The profile of one of the cutmarks identified in a rib—asymmetrical, and with step-like striations on the shallower side of the cutmark, seems to conform to the use of bamboo knives for dismembering [68, 69]. The use of bamboo tools for processing human remains has been reported from a number of locations in Southeast Asia, New Guinea, the Torres Strait, and Polynesia [69–74]. In Tron Bon Lei, where imported obsidian, abundant fine-grained basalt, and chert were available [19], the use of bamboo knives for dismembering of human remains could be related to the specialised usage of these implements for mortuary ritual purposes. However, due to the scarcity of cutmarks available for analysis, this hypothesis remains unconfirmed. Further analysis will look at cutmarks produced experimentally by different materials (i.e. obsidian, basalt, chert and bamboo). Nevertheless, our analysis supports the use of laser scanning confocal microscopy as a useful tool in characterising cutmark profiles to identify cutting materials from bone surface modifications.

As part of the burial rites, a large haematite nodule was placed over the body, with some elements displaying red pigmentation. Regarding the reddish staining on the upper dentition, further investigation is required to discern whether this coloration is also the result of the intentional application of a red pigment, presumably related to the burial rites, or alternatively, is a consequence of betel nut chewing during the individual’s life.

The timing of the application of pigment to the rib and vertebrae bodies is uncertain, although the presence of several rocks and cobbles with ochre coating associated with the burial suggest that ochre processing might have been part of the burial rites performed during deposition of the body. That all eight large rocks associated with burial had ochre on them, many of which were irregularly shaped beach cobbles and rough clasts, suggests the application of ochre was part of the rite rather than ochre processing for other reasons. The four cobbles with cupules on them are noteworthy, as they could have been used for cracking *Canarium* spp. nuts, a practice observed in the field and confirmed by local collaborators.

### Tron Bon Lei in the context of Southeast Asian mortuary practices

The three burials documented in Tron Bon Lei provide additional data to discuss mortuary practices in Mainland (MSEA) and Island Southeast Asia (ISEA) from the late Pleistocene to the middle Holocene. For a description of MSEA and ISEA burials discussed in this section, as well as references, we refer the reader to the S1 File and S1 Table.

**Fig 12:**
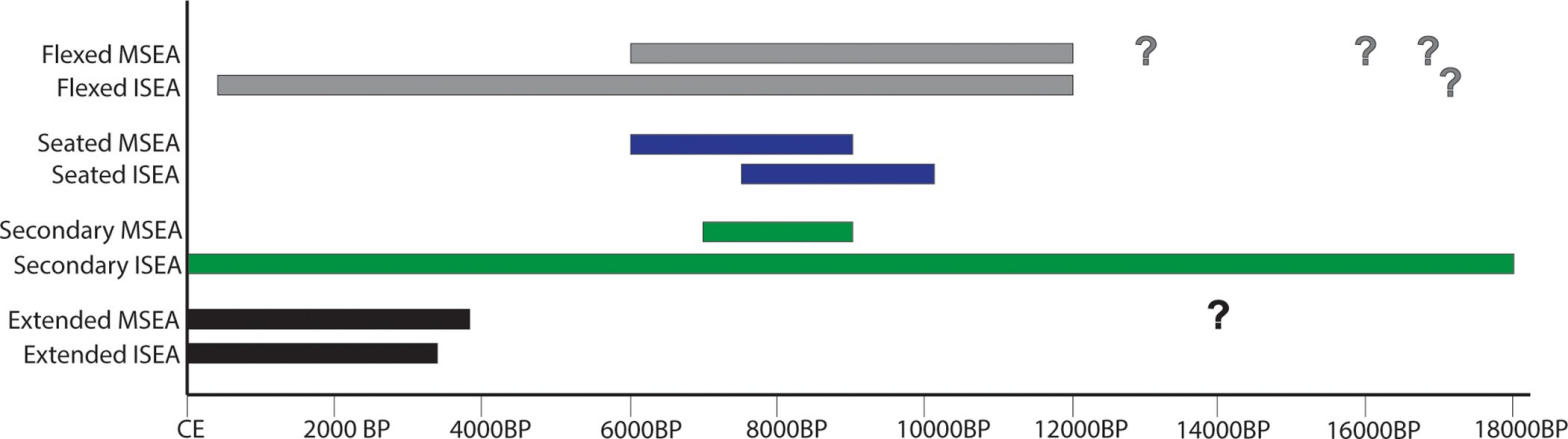
Chronology of burial types. Bar chart showing the chronology of the different burial types discussed in this paper. Question marks indicate possible burials, which chronology or burial type is uncertain (see discussion text).

The oldest burial excavated in Tron Bon Lei (TLB-1), dated to 11.1–12.6kya cal BP, is a primary flexed burial of an adult female, representing a burial type commonly adopted in MSEA and ISEA during the late Pleistocene (Fig 12). Examples of primary flexed burials dated to c.11kya cal BP have been documented in Hang Cho (Vietnam), Ban Rai (Thailand), GK1 from Gua Kajang (Malay Peninsula), and Goa Braholo, burial 1 (Java, Indonesia). Other flexed burials that might precede TLB-1 by a few thousand years, were documented in Moh Khiew (9.2–13.1kya cal BP; Thailand), Tham Lod, burial 2 (c.16.2–16.7kya cal BP; Thailand), and BHL6 from Goa Braholo (c.14.9–17.3kya cal BP; Java). However, the reliability of some of the dates associated with these burials is questionable. In GK1 from Gua Kajang, the published date (10,820±60 BP) was obtained from a freshwater shell from the burial context [75] and freshwater species are known to yield anomalously old radiocarbon ages, due the freshwater reservoir effect [76]. Other dates are derived from charcoal recovered from the archaeological layer into which the burials were interred and thus may be older than the burial context [52]. Currently, the only direct date for these burials comes from the burial in Hang Cho, where one of the skeletal elements was dated to 9,259±260 BP (c.11kya cal BP).

The three radiocarbon dates published from TLB-1 were obtained from two charcoal samples directly associated with the burial (from under the skull, and inside the eye socket), as well as a direct date on one of the fish-hooks found on the neck of the individual [8, 9]. Even though the direct U-Th date is anomalous and thought to have returned an estimate that is too old, we consider that the similarity of the radiocarbon ages obtained from the burial context support the c.12kya cal BP chronology for this burial.

Even allowing for some issues with the dating, it would appear that the practice of primary flexed burial was very widespread from the terminal Pleistocene. Flexed burials remain in practice in ISEA until the late Holocene, with examples in Pain Haka (2.1-3kya cal BP; Flores) and Jareng Bori (c.400 cal BP; Pantar). However, this burial position seems to have been abandoned in MSEA thousands of years earlier, with the last examples coming from the Hoabinhian burials in Gua Cha (c.6kya cal BP; Malay Peninsula). The survival of flexed burials in the southern Wallacean islands might indicate a regional practice, which persisted even after new burial practices were integrated following Austronesian dispersal, but was replaced by extended and secondary burial in islands to the north such as Borneo, Sulawesi and Northern Maluku.

The oldest evidence for seated burials in the region consists of the middle burial documented in Tron Bon Lei (DE B2), dated by associated charcoal to c.9.5–10.2kya cal BP, although attempts to obtain a direct date from this burial are underway. Seated burials do not appear to be as commonly adopted as flexed or secondary practices, with the mid-Holocene burials from the West Mouth of Niah cave (c.7.5-8kya cal BP) being the only other examples in ISEA. In MSEA, seated burials appear limited to the northern areas included in this study, with the earliest examples documented at Huiyaotian (Guanxi, China), where sixteen of the thirty-five burials are identified as seated/squatting and dated by association to c.9kya cal BP. This practice was recorded in the extensive cemetery of Con Co Ngua (c.6kya cal BP; Vietnam), and in the individual from Tam Pong (Laos; c.6kya cal BP).

The low number of sites where seated burials have been documented coupled with the large number of seated burials in most of these sites, raises questions about the short duration and limited geographic representation of this form of burial in Southeast Asia. Several taphonomic factors, such as post-depositional anthropic disturbances, gravity, sedimentation rates, funerary space and practices, and dissociation processes resulting from soft tissue decomposition, can modify the original burial position [67]. In this sense, and as exemplified by the DE B2 burial from Tron Bon Lei, the thorax can succumb to gravity, moving from a vertical to a supine position. Furthermore, the lower extremities can move together toward either side or fall outward individually. As such, some tightly flexed burials, where the legs are folded towards the chest, and interpreted as a foetal position, could indeed correspond to seated burials, where the full body, wrapped or inside some organic container, has moved by gravity while infilling was incomplete. Even though this hypothesis is difficult to test in old excavated burials, we anticipate that archaeothanatology approaches in recently discovered burials or future excavations will assist in the identification of the original burial position, and the effect of taphonomic processes in secondary body deposition.

The most recent Tron Bon Lei individual, E16b, is a secondary burial. Secondary burials have a long tradition in ISEA, However, in MSEA secondary practices are only recorded in the southern China sites of Huiyaotian and Dingsishan (c.7-6kya cal BP; Guanxi). The Liang Lembudu woman (c.16-18kya cal BP; Aru Island) is the earliest evidence of this form of burial in the region. In Ille Cave (c.9-9.6kya cal BP; Philippines), a secondary burial of a female displays evidence of dismembering, as in Lembudu. Secondary practices are also recorded in Niah Cave beginning at c.7.5kya cal BP. The E16b individual from Tron Bon Lei (c.7.5kya cal BP) shows dismembering marks and a deliberate selection of the skeletal elements to be buried, with the removal of the long bones and mandible. This intentional practice is also observed in the only other site thus far excavated in Alor, Makpan, where a secondary burial of approximately the same age (c.8kya cal BP) was recovered [7]. Human remains identified from the adjoining Tron Bon Lei shelter in square C (TLC-1; c. 7-17kya cal BP) comprise a distinct deposition of skull, upper cervical vertebrae (C1-C5), and clavicles, with fragmentary vertebrae, ribs, carpals, metacarpals, and hand phalanges, which could also be interpreted as a secondary burial with absence of long bones [9]. Several cobbles were recorded surrounding and over these remains.

Secondary practices are common in late Lapita contexts, such as the Teouma (Vanuatu) cemetery, dated to c. 3kya cal BP, with removal and secondary deposition of specific elements [4]. Manipulation of the remains has also been recorded in northern Maluku sites (c. 2kya cal BP), with secondary jar burials in historic sites from the Towuti-Routa region [77]

Secondary practices appear to be a long-standing tradition in ISEA, coexisting with extended burials which appear c.4kya cal BP, and are usually linked to the Austronesian expansion. However, two early extended burials have been reported from Tham Lod (burial 1; c. 14kya cal BP) and Song Keplek 5 (SK5; c. 8kya cal BP). Nevertheless, the burial position of the Tham Lod individual is difficult to corroborate, based on the low number of bones recovered and the original diagram, while the burial was indirectly dated on a charcoal sample from near the tibia [78]. As such, the exact dating and burial type of the Tham Lod burial 1 is uncertain. Furthermore, a recent direct date obtained from SK5, dating the burial to 3.1–3.5kya cal BP, rejects its previously suggested antiquity [52, 53, 79].

In addition to treatment of the remains, the analysis of associated grave materials informs on the cultural behaviour of past communities. All the burials from Tron Bon Lei show deliberate deposition of large water-rolled cobbles and angular rocks, with some of them used as “grave pillows”. As noted above, several of the cobbles also have evidence of deep pitting or cupules corresponding with their likely use as *Canarium* spp. nut cracking mortars. Similar pitted pebbles are reported from the preceramic layers at Tanjung Pinang (southern Morotai, Maluku), stated by local informants to have been used ethnographically for cracking *Canarium* nuts [80]. The cobbles are also mostly coated in ochre, with distinct deposition of haematite pigment in the crenulations and on surfaces. The intentional deposition of water-rolled cobbles, slabs and rocks is recorded in several sites from MSEA and ISEA, with inclusion of haematite nodules and ochre staining on the skeletal remains (S1 Table).

In Tron Bon Lei, the old adult female burials (TLB-1 and DE B2) include subsistence implements, such as a grindstone and fish-hooks. The inclusion of these elements into female burials suggest the importance of these daily tools in the lifeways of female individuals within the community. The richness of the burials, particularly TLB-1, in combination with the advanced age of the females at death, suggests a high degree of status in life. Excluding the individuals from Tam Hang (Laos) where no information about mortuary practices is available, other female burials are also associated with lithic implements: an anvil, hammerstone and flakes in Gua Kajang (GK1, Malay Peninsula), flakes and cores in burial 2 from Tham Lod (Thailand), and three stone artefacts in burial 1 from Moh Khiew (Thailand).

Regarding gender differences in the treatment of the deceased; the interment of females seems more prevalent in the Late Pleistocene sites reviewed, with eleven of the fifteen sexed burials documented in MSEA and ISEA, comprising women (S1 Table). The female/male ratio is more balanced from the early Holocene onward, with no clear sexual division in the mid-late Holocene large cemeteries of Guangxi province (China), northern Vietnam, and Pain Haka (Flores, Indonesia). These gender differences between the Pleistocene and Holocene burials could reflect the significant position of females in past communities in the region.

A significant caveat when reviewing mortuary practices in MSEA and ISEA is where and how the preservation of burial sites occurred. The burgeoning of burial sites from the early Holocene onwards could reflect demographic and settlement changes with larger populations, and increased sedentism, resulting in a greater concentration of interments near settlements i.e. cemeteries. However, the differences in the number of burials could also be related to poor preservation of earlier interments, and a focus on the excavation of cave sites to counteract poor preservation in tropical open-air sites. As such, the large cemeteries documented in southern China and northern Vietnam are open-air contexts in cooler subtropical regions, where preservation of skeletal remains might be more favourable. Analysis of histological degradation on subtropical environments to understand bone diagenesis, does not seem to identify great variations in the degree of bioerosion in subtropical and tropical environments, with high intensity of bacterial attack relative to temperate-cold climates in both [81]. However, sufficient experiments and research have not yet been performed to confirm this hypothesis. An increase in survey and excavation of open-air locations in ISEA could provide more data to evaluate whether differences in the number of burials identified from the Holocene results from larger populations, greater sedentism, or is a consequence of bone diagenesis processes.

## Conclusions

The Tron Bon Lei rockshelter comprises a significant site to interpret burial practices in the Lesser Sunda Islands. The three burials described in this paper comprise evidence of diverse burial practices from the terminal Pleistocene to the mid-Holocene.

Commonalities between Mainland and Island Southeast Asian mortuary treatments beginning in the Late Pleistocene, with a common custom of flexed burial as seen in Tron Bon Lei (TLB-1), suggest transmission of ideas. This may be due to migrations or cultural transmissions related to maritime exchange, as demonstrated by the import of obsidian from surrounding islands beginning at around the same time. However, regional variations in grave associations and anthropogenic modifications, and a discontinued adoption of specific burial practices (i.e. secondary and seated burials), could also indicate local emergence of these practices. Improved chronological accuracy and detailed archaeothanatological studies are needed to better characterise patterns in mortuary treatments across this region.

Further research in aspects such as biomolecular anthropology, diet practices based on dental macro- and microwear evidence, or the type of tools (i.e. bamboo versus lithic) used in burial rites will allow us to gather more data, complementing the valuable information offered by the study of burial practices. These future efforts will provide us deeper insights to interpret the lifeways of communities inhabiting Mainland and Island Southeast Asia during the Pleistocene and Holocene.

## Supporting information

S1 File Burial practices in Mainland and Island Southeast Asia during the Pleistocene and Holocene. This file provides a review of published burials from Mainland and Island Southeast Asia dated from the Pleistocene until the mid-Holocene, with relevant late Holocene examples included. The file is divided according to regions, with burials introduced in each region following chronological order.
(PDF)

S2 File Description of skeletal elements from Tron Bon Lei. Detailed description of the skeletal elements recovered in the three burials presented in this manuscript.
(PDF)

S1 Table Burial sites in Mainland and Island Souteast Asia. Sites mentioned on the review of mortuary practices in Mainland and Island Southeast Asia. Numbers in brackets correspond to site number in Fig 1. Uncalibrated dates available calibrated by OxCal v.4.4. [13]. IntCal20 [15] used for charcoal samples. Shell samples were calibrated using the Marine20 [14] calibration curve. Abbreviations: Y = yes; N = no; YA = young adult; MA = mid-adult; A = adult; OA = old adult; M = male; F = female;? = uncertain/unknown; NA = not applicable.
(CSV)

S2 Table Results of the Bayesian model of occupation for Tron Bon Lei, square B. Bayesian model produced by OxCal v.4.4. [13]. An U(0,50) mixed calibration curve with IntCal20 [15] and SHCal20 [16] curves used for charcoal samples—as recommended for the Inter-Tropical Convergence Zone [16, 17]. Marine shell samples were calibrated using the Marine20 [14] calibration curve. For our charcoal dates we applied the Charcoal Plus t-type Outlier Model with a prior outlier probability of 10% for each date, which is specifically designed to account for the inbuilt age of charcoal (i.e. old wood effect) while also allowing for some stratigraphic movement in an archaeological context [82, 83]. The General t-type Outlier Model with a prior outlier probability of 5% was used for the remaining dates, following commonly used modelling procedures for general archaeological dates [82].
(CSV)
