# Supplementary material for: Talking Dead. New burials from Tron Bon Lei (Alor Island, Indonesia) inform on the evolution of mortuary practices from the terminal Pleistocene to the Holocene in Southeast Asia
Source: PLoS One. 2022 Aug 24;17(8):e0267635. doi: 10.1371/journal.pone.0267635 (PMC9401180; doi:10.1371/journal.pone.0267635)
Supplement: S1 File — This file provides a review of published burials from Mainland and Island Southeast Asia dated from the Pleistocene until the mid-Holocene, with relevant late Holocene examples included. The file is divided according to regions, with burials introduced in each region following chronological order. (PDF) [file pone.0267635.s001.pdf]

S1 File. Burial practices in Mainland and Island Southeast Asia during the Pleistocene and Holocene

Mainland Southeast Asia (MSEA)

Several sites in MSEA have yielded burials dated to the terminal Pleistocene, although dating accuracy is sometimes controversial (Fig. 1; S1 Table; see discussion). From the early Holocene onwards, the number of burial sites and individuals recovered in MSEA sites increases drastically, with a large number of internments documented in open air sites. Here we present a review of the burials recovered in MSEA sites following a geographical order, starting on the northern sites from south China and finishing with the records available for the Malay Peninsula. For each region, we follow a chronological order for the description of the interments.

Guangxi region (south China)

Research on the Guanxi region has unveiled a large number of burials dated to the early Holocene onward. At Dingsishan, 331 burials spanning c.9-7 kya BP were recovered, with the main burial types identified being flexed and secondary/dismembered burials. [1,2]. At Huiyaotian (c.9kya cal BP), 60 burials, mostly comprising adult individuals with burial types including flexed, squatting, and secondary burials, were recovered [3]. No manuports or grave goods were identified in either of these sites.

Vietnam

In northern Vietnam, Han Cho cave contains a burial directly dated to c.11kya cal BP [4]. The individual, an old female, was buried in a supine flexed position, with the head towards the east and devoid of grave goods [4].

In Thanh Hoa province, an extended supine burial dated to the early Holocene was identified in Mai Da Nuoc [5]. This burial, assigned to an old male, has been dated to c. 10-8kya BP based on

associated Hoabinhian artefacts, although absolute dates are unavailable. No grave goods are reported from the burial.

Ninety-six burials have been recorded in Con Co Ngua (c.6kya cal BP), in northern Vietnam, commonly interred in a tightly flexed squatting position [6,7]. The site of Man Bac (3.7-3.9kya cal BP), the more extensively studied cemetery in northern Vietnam, yield over 80 extended burials, which include child and adult internments of males and females [7]. Grave associations include rocks and globular pots in the majority of the burials.

Laos

In northern Laos, the Tam Hang site yielded a minimum of 17 individuals, dated to c.17kya cal BP based on a direct date from one burial (THS10) [8]. There are no available dates for the rest of burials. Information regarding body positioning is limited to the original description of the findings [9], where three adults were buried in supine position (no detail regarding flexed or extended), another adult buried extended lying on its side, and two children squatting against the wall, with the latter three individuals hypothesized to be victims of a landslide [9,10]. Recent bioskeletal profiling of the ten individuals available for analysis from the original excavation indicates the majority are females, with all the individuals identified as adults [11].

A well-preserved flexed burial was recorded in Pha Phen, dating to 7.8kya cal BP [10]. It comprises an adult male lying on its left side, with no associated grave goods. In Tam Pong (6kya cal BP), a young adult individual was recorded as seated/squatting, lying on the back [9,10].

Thailand

Burials dated to the late Pleistocene-Hoabinhian cultural period in northern Thailand include two individuals excavated in Tham Lod (dated to 16.2-16.7 kya cal BP and 13.8-14 kya cal BP, respectively) and a burial from the Ban Rai site (11-11.2kya cal BP) [10]. The oldest burial from Tham Lod comprises an adult interred in flexed position on its side, while the second corresponds to an adult allegedly buried in supine extended position, although the skeleton was less that 50 percent complete [12,13]. Both

burials were associated with manuports, such as large cobbles placed in a circle (burial 2) and a hammerstone on top of the femur (burial 1). The individual from Ban Rai was identified as a male with an estimated age at death of 45-50 years. The body was interred in a supine flexed position [12,13]. In southern Thailand, the site of Moh Khiew yielded several human remains, with the best known (MKC91 B2) an adult flexed burial, dated by associated charcoal to the terminal Pleistocene-early Holocene (9.2-13.1 cal BP) [14,15]. A second individual (MKC91 B1) was dated to c.25kya BP based on charcoal associated with the burial, although the reliability of this date is unclear [15]. A remarkable aspect of both of these burials is the identification of manuports within the burial contexts, consisting of large slabs or rocks, and stone artefacts (i.e. hammerstone and flakes) intentionally positioned over the skeletons.

A burial in Ban Tha Shi (Thailand), directly dated to c.8kya cal BP, is identifed as a male buried on its right side, with the head absent, and the lower and upper limbs flexed, with the hands towards the face [16].

Cambodia

A complete mortuary analysis has been published for the extended burial at Laang Spean, dated 3.4-3.6 cal BP [17]. It comprises the complete skeleton of an adult male, with associated grave goods and evidence of opening and re-sealing after decomposition of the body [17].

Malay Peninsula

The Gua Kajang site yielded two burials, dated by associated shell to 8-8.6kya cal BP (GK2) and 11.9-12.6 kya cal BP (GK1) [18]. Due to modern disturbance, body positioning of GK2, as well as the integrity of the associated grave goods (stone tools, animal bone, shell and manuports) is unclear. The GK1 burial consists of an incomplete skeleton of an adult male, while the presence of articulated elements indicates a flexed body position. Several animal bones, stone artefacts (including an anvil and hammerstone) and riverine shells were recorded within the burial context and are interpreted as grave goods [18].

In Gua Gunung Runtuh, the so-called “Perak man” was excavated in 1990. Dated c.10.8-11.6kya cal BP by associated freshwater shell, the Perak man comprise a primary highly flexed complete skeleton, with both legs folded up to the chest in a foetal position and the upper body in supine position [19]. The individual was identified as an old adult male (over 40 years old), displaying pathologies on the left hand phalanges, zygopodia and vertebral column [20]. The associated grave goods consist of stone artefacts (including a slab with red haematite and a pebble tool), animal bones and freshwater shells [19]. Another early Holocene burial was documented in Gua Teluk Kelawar, dated on associated shell to 8.8-9.2kya cal BP [19]. The GTK1 individual is an old adult female, buried in a highly flexed foetal position, laying on the left side. Associated grave goods include stone tools, a large number of animal bones and freshwater shells [19]. Gua Peraling contained four individuals. The best preserved of these skeletons (Gua Peraling 4) consists of a semi-complete skull, numerous ribs and vertebrae fragments, a fragmented pelvis and partial long bones from a young adult [21]. The internment, dated to c.6-8kya cal BP, can be interpreted as a primary flexed burial with significant post-burial disturbance, or an unusual secondary burial.

At Gua Cha, flexed burials dominate the Hoabinhian period at the site (c. 6kya cal BP; 15 burials), with a change to extended burial in the Neolithic period (c. 3kya cal BP; 23 burials). Grave goods, such as jewellery and pots are common in the Neolithic burials, while associated material was sparse in the oldest internments [22].

Island Southeast Asia (ISEA)

East of the Wallace Line, burials dated to the terminal Pleistocene are rare, with a drastic increase in the number of burials recorded from the early Holocene (Fig. 1; S1 Table From the late Holocene (c.4kya cal BP) onwards, grave practices and the material associated with burials seems to gravitate towards supine extended internments. Nevertheless, a variety of mortuary practices continue to be observed in sites at Sulawesi, northern Moluccas, and the Lesser Sunda Islands.

Our description follows a West-East direction, beginning in Java and finishing in Aru Island, with a separated section for the Lesser Sunda Islands. The burials recorded in each islands are described

in chronological order, from oldest to earliest. Regarding historic sites, in this review we include only those sites with mortuary practices relevant to the internments in Tron Bon Lei, due to geographic proximity.

Java

Several burials dated to the Late Pleistocene and early/mid-Holocene have been recorded in sites along the south coast of Java (Indonesia), with the Gunung Sewu cave complex comprising a node of archaeological sites, such as Goa Braholo, Song Terus and Song Keplek, with evidence of recurrent human occupations [15,23,24]. In Goa Braholo (BHL), with occupations dated from 33,000 to 3000 years before present, at least seven individuals and a large number of isolated remains were recovered. The oldest of these internments are the flexed burials BHL6 (14-17kya cal BP) and BHL1 (10-12kya cal BP). BHL1 was assigned to a young adult male, with a west-east orientation, in a flexed supine position and with rocks intentionally placed over the feet and torso of the deceased [15]. BHL6 consists of a primary highly flexed burial, laying on its right side, with the knees flexed upwards beside the body and flexed arms [15]. The other five burials originally identified in Goa Braholo are dated to 8-10kya BP based on stratigraphic correlations, with only one of these burial (BHL2) yielding a more precise date of 9.8-10.2kya cal BP from a charcoal sample associated with the individual. BHL2 comprise a secondary burial, with presumably selective removal of skeletal elements after the original deposition of the skeleton. The skeletal elements recovered include the cranium, mandible, incomplete pelvis, and one cervical vertebra [15,25]. The elements are dissociated from each other, disarticulated, although non-randomly buried. Evidence of burial preparation was identified based on the presence of a bed of ashes below the bones, which are not burnt [15,25]. The individual was identified as a young adult female.

The cave of Song Terus yielded isolated human remains, with a date on marine shell associated with the skeleton of 10.2-10.7kya cal BP. The individual, an adult male with extreme dental wear, was buried in primary highly flexed position, resting on the right side inside a natural “niche” on the north wall of the cave. The back was resting against the wall, with several limestone rocks placed in front of

the skeleton, sealing the burial [15,25]. In addition to the rocks, other materials associated with the burial included a large chert artefact, a bone point, and a large number of faunal remains [15].

Song Keplek yielded at least five individuals, with two burials dated to the early/mid-Holocene [15,25]. SK4, with an estimated date of c.6kya cal BP based on associated charcoal, represents a highly flexed burial. The individual, identified as a young adult female, was buried on its right side, with extremely flexed legs, feet in hyper-extension and arms folded up along the body, a body positioning that has been interpreted as likely resulting from the use of bonds or internment in a narrow burial pit. The individual was identified as an adult female, with an estimated age at death of 30 years old [15,25]. SK5, dated on associated charcoal to 7.5-8.2kya cal BP, with a recent direct date of 3.1-3.5kya cal BP, comprises an extended primary burial of an articulated almost complete skeleton [15,25,26]. Significant post-mortem displacement of skeletal elements, which affected both clavicles, the left humerus and ulna, both femora, both patellae, the calcanei and metatarsals, indicates partial voids or soil pressure; the latter further supported by the fragmentation of the pelvis and hyperextension of the feet. Grave associations included rocks placed over the body and a macaque cranium under the fingers of the right hand [15,25]. Our personal observations on the published material identify this individual as an adult, although no sex assessment is available.

In East Java, the site of Song Gentong II yielded a fragmentary burial with an uncertain Holocene date [15]. This adult individual was buried on the left side, with a large rock and mortar associated with the skeleton, and haematite pigment spread over and around the body [15]. The site of Gua Lawa yielded two burials, one of them identified as a cremated burial of an adult individual. The burial denotes an intentional selection of skeletal elements, including cranial fragments, and fragments of ribs and unidentified long bones [15,25]. Unfortunately, the chronology of this burial is uncertain, being broadly assigned to an early Holocene phase [25,27].

In West Java, the site of Gua Pawon yielded burials dated to the early and mid-Holocene. The oldest (PAW4), directly dated to c. 10.2-11.2kya cal BP, comprises a flexed burial, with the head supported by a sandstone block [26]. A highly flexed burial (PAW3), dated c.7.7-8.4, had a rock placed on the chest [28]]. The partial burials PAW1 and PAW2, dated by stratigraphic association to c.6.2-6.9kya cal BP, were stained with red pigment, possibly haematite [26,28].

Borneo (Kalimatan)

The West Mouth of the Niah Cave complex (Borneo) yield a large concentration of burials, ranging from the early Holocene to the late Holocene and displaying a variety of burial practices, including primary flexed and seated/squatting burials, decapitated individuals, and secondary burials cremated and unburned [29–32]. The oldest of the directly dated burials (B155; c.8.3-9.1kya cal BP) comprised a flexed internment of an adult female, whose skull has been intentionally removed [31]. Another adult female was buried in a seated/squatting position (B147), with a direct date on bone of c. 7.6-8kya cal BP [31]. Burial B92, dated to c.7.3-7.6kya cal BP comprise a secondary burial of a child (7-12 years old), with skull removed [31]. Grave associations for the early Holocene Niah burials include cobbles, some of them used as mortars to grind ochre and plant material, bone tools and a rhinoceros radius placed as a "grave pillow"; with some individuals displaying ochre application to the skull [30–33].

An extensive dating program, as well as a detailed mortuary study of the 'Neolithic' burials from the West Mouth of Niah cave (Sarawak, Borneo) permit an assessment of shifts in burial practices during the late Holocene. Several flexed burials are dated to around 3.3-3.5kya BP, with a change to extended supine burial after 3.3kya BP. This change can also be observed in the layout of a formalized and structured cemetery, with rows of extended burials, in wooden coffins and wrapped in shrouds [34]. Grave associations included pots, stone adzes and grinders, beads, basketry and textiles [34]. Secondary burials are the dominant rite after c.3kya BP, where extended burials were reopened, skeletons manipulated, and skulls removed and curated, before re-burial. By c.2.7kya BP, cremation is adopted as the main burial form, marking a spatial reorganisation of the cemetery [34]. The final main phase of the cemetery (c.2.2-2.4kya BP) witnessed a reversion to non-burnt secondary and to primary extended burials.

Dated by stratigraphic association to the mid-Holocene, three flexed burials have been documented at Kimanis and Gua Tengkorak, in the east of Borneo (Indonesia) [33,35].

Sulawesi

In Sulawesi, a primary flexed burial was documented at Leang Panninge, dated c.7.2-7.3kya cal BP [36]. This individual, identified as a young adult female, was buried in a flexed position and covered with large cobbles [36](Carlhoff et al. 2021).

Bulbeck's extensive revision of the mortuary practices in the Towuti-Routa region (Southeast Sulawesi, Indonesia), including comparisons with other ISEA sites with similar chronologies, highlights the diversity of burial forms in the regions from the first millennium CE [37]. In several of the burial sites included in this comparative study, extended burials were accompanied by secondary inhumations, jar burials and some flexed burials (Table 12)[36]. Grave associations included pots, shell adzes and ornaments, polished stone artefacts, glass beads, and metal items.

Philippines

In Ille cave (northern Palawan, Philippines), five cremated burials dated to the early Holocene have been recovered [32,38]. One, B758 (c.9-9.6kya cal BP) has been identified as a partial skeleton of a young-mid adult female. The skeletal elements show evidence of post-mortem breakage, with cut and scrape marks near the joints and on the surface of long bone, indicating dismembering and defleshing. The remains were placed in an organic container of wrapping, that was buried at the entrance of the cave [32].

A highly flexed burial of a young adult, dated to c..4.6-4.8kya cal BP, has been excavated at Bubog I (Ilin, Philippines) [27]. No evidence of grave associations or body modifications was recorded.

Moluccas

This diversity is equally identified in human burials excavated from several rockshelters in the Northern Moluccas [39]. In these sites, an extended burial from Golo cave (Gebe Island) dated to 1.4-2.3kya cal BP has a similar chronology to the secondary skull burial from Uattamdi (Kayoa island), dated to 1.8-1.9kya cal BP, while secondary burials and cremations are common from the BP/CE

junction into the first millenia CE, with the ten individuals excavated in Tanjung Pinang (Morotai) representing secondary skull burials, all assigned to males [39].

Aru Island

The earliest evidence of intentional burial East of the Wallace Line comes from Liang Lembudu (Aru Island), dated by associated charcoal to 16-18kya cal BP [40]. Although at that time Aru Island was part of the Sahul continental shelf, it is included in this review due to its proximity to Wallacean islands to the west. The Liang Lembudu individual has been identified as an adult female interred in a secondary burial, with evidence of dismemberment in the form of cut marks on the upper limbs, and selective removal of elements, such as hands and feet. The arrangement of skeletal portions suggests that the burial practice involved the internment of skeletal elements in bundles, presumably wrapped in some organic material, with the individual placed in a seated position [40]. A large flat boulder was identified during excavation, partially covering the burial, and interpreted as originally placed to cover the internment [40]. The "Lembudu woman" comprises the earliest evidence of intentional removal of skeletal elements, dismembering and burial recorded to date. Another secondary burial was reported from Liang Nabulei Lisa, dated to 10-12kya cal BP on associated charcoal, comprises burnt and unburnt commingled remains from at least four individuals, including two children [41].

Lesser Sunda Islands

The oldest burials documented in the Lesser Sunda Islands come from Alor Island. Besides the burials excavated in Tron Bon Lei presented in this paper, a child burial (5-8 years old) was excavated at Gua Makpan [42]. The long bones were intentionally removed, even though some of the unfused epiphyses are preserved. The articulation of some skeletal elements, such as the feet, combined with the absence of cutmarks on the preserved joints suggest that the burial could be interpreted as a delayed primary burial, with the skeleton being exposed prior to internment, or a secondary burial. Additionally, the anatomical association of the smallest bones from the feet (i.e. phalangeal epiphyses) suggests the use of wrapping material or the internment of these elements before complete decomposition of the

connective soft tissue [42]. A cobble coated in red ochre was intentionally placed below the head of the individual, resulting in the flexion of the cervical vertebrae.

Burials in Pain Haka (Flores, Indonesia), dated c.2.1-3kya cal BP, illustrate diverse mortuary practices, with flexed and seated burials, and evidence of dismembering and intentional removal of skeletal elements, although the majority are supine extended burials [43]. Some of the burials contained commingled remains, representing more than a single individual. Burial containers include organic wrapping around the corpse as well as pottery jars. Although the palaeodemographic analysis of the cemetery is still underway, no age or sex segregation of the burials was initially reported. Grave associations include pottery (as burial jars or placed alongside the skeletons), stone adzes, shell adzes and ornaments, large coral stones, a pig tooth and a stingray barb [43].

At Jareng Bori (Pantar, Indonesia) a flexed burial dated to c.400 cal BP was excavated, with no associated material in the grave, but evidence of dental filing on the anterior teeth [44].

References S1 File.

1. Xianguo F. The Dingsishan site and the prehistory of Guangxi, south China. Bull Indo-Pac Prehistory Assoc. 2002;22:63–72.

2. Zhu S, Li F, Chen X, Fu X, Hu Y. Subsistence and health in Middle Neolithic (9000–7000 BP) southern China: new evidence from the Dingsishan site. Antiq Q Rev Archaeol. 2021;95(379):13–26.

3. Matsumura H, Hung H, Zhen L, Shinoda K. Bio-anthropological studies of early Holocene hunter-gatherer sites at Huiyaotian and Liyupo in Guangxi, China. National Museum of Nature and Science Tokyo; 2017.

4. Matsumura H, Yoneda M, Dodo Y, Oxenham M, Cuong NL, Thuy NK, et al. Terminal Pleistocene human skeleton from Hang Cho cave, northern Vietnam: implications for the biological affinities of Hoabinhian people. Anthropol Sci. 2008;116(3):201–17.

5. Cuong NL. Two early Hoabinhian crania from Thanh Hoa province, Vietnam. Z Morphol Anthropol. 1986;77(1):11–7.

6. Nguyen Viet. The Da But culture: evidence for cultural development in Vietnam during the Middle Holocene. Indo-Pac Prehistory Assoc Bull. 2005;25:89–93.

7. Oxenham M, Matsumura H, Dung NK, editors. Man Bac: the excavation of a Neolithic site in northern Vietnam. The Biology. Canberra: ANU E Press; 2011. (Terra Australis).

8. Demeter F. Histoire du peuplement humain de l’Asie extrême-orientale depuis le pléitocène supérieur récent. Paris 1; 2000.

9. Fromaget J, Saurin E. Note préliminaire sur les formations Cénozoiques et plus récentes de la chaîne Annamitique septentrionale et du Haut-Laos (stratigraphie, préhistoire, anthropologie). Impr. d'Extrême-Orient; 1936.

10. Tayles N, Halcrow SE, Sayavongkhamdy T, Souksavatdy V. A prehistoric flexed human burial from Pha Phen, Middle Mekong Valley, Laos: its context in Southeast Asia. Anthropol Sci. 2015;123(1):1–12.

11. Shackelford L, Demeter F. The place of Tam Hang in Southeast Asian human evolution. Comptes Rendus Palevol. 2012;11:97–115.

12. Pureepatpong N. Recent investigations of early people (late Pleistocene to early Holocene) from Ban Rai and Tham Lod rock shelter sites, Pang Mapha district, Mae Hongson province, Northwestern Thailand. In: Bacus EA, Glover IC, Pigott VC, editors. Uncovering Southeast Asia's past: Selected papers from the 10th international conference of the European association of Southeast Asian archaeologists. Singapore: NUSS Press; 2006. p. 38–45.

13. Shoocongdej R. Late pleistocene activities at the Tham Lod rockshelter in highland Pang Mapha, Mae hong Son province, northwestern Thailand. In: Bacus EA, Glover IC, Pigott VC, editors. Uncovering Southeast Asia's past: Selected papers from the 10th international conference of the European association of Southeast Asian archaeologists. Singapore: NUSS Press; 2006. p. 22–37.

14. Pookajorn S. New perspectives for Palaeolithic research. In: Semah F, Falguères C, Grimaud-Herve D, Semah AM, editors. Origin of settlements and chronology of the palaeolithic cultures in Southeast Asia. Paris: Semenanjung; 2001. p. 167–87.

15. Détroit F. Origine et évolution des Homo sapiens en Asie du Sud-Est: Descriptions et analyses morphométriques de nouveaux fossiles. [Paris]: Muséum National d´Histoire Naturelle; 2002.

16. Zeitoun V, Auetrakulvit P, Forestier H, Zazzo A, Davtian G, Nakbunlung S, et al. Discovery of a Mesolithic burial near the painter rock-shelter of Ban Tha Si (Lampang province, Northern Thailand): Implications for regional mortuary practices. Comptes Rendus Palevol. 2013;12:127–36.

17. Zeitoun V, Forestier H, Sophady H, Puaud S, Billault L. Direct dating of a Neolithic burial in the Laang Spean cave (Battambang Province, Cambodia): First regional chrono-cultural implications. Comptes Rendus Palevol. 2012;11:529–37.

18. Goh HM, Saidin M. The prehistoric human presence in Gua Kajang: ancient lifeways in the Malay Peninsula. J Malays Branch R Asiat Soc. 2018;91(3):1–18.

19. Majid Z. The excavation and analyses of the Perak man buried in Gua Gunung Runtuh, Lenggong, Perak. Perak Man Prehist Skelet Malays Penerbit Univ Sains Malays Penang. 2005;1–32.

20. Jacob T, Soepriyo A. A palaeoanthropological study of the Gua Gunung Runtuh skeleton. In: Majid Z, editor. The Perak Man and other prehistoric skeletons of Malaysia. Malaysia: Penerbit Universiti Sains; 2005. p. 33–50.

21. Bulbeck D, Taha A. A description and analysis of the Gua Peraling human remains. In: Majid Z, editor. The Perak Man and other prehistoric skeletons of Malaysia. Malaysia: Penerbit Universiti Sains; 2005. p. 311–44.

22. Bulbeck D. The Gua Cha burials - concordance, chronology, demography. Duckworth Laboratory: Cambride University; 2001.

23. Simanjuntak T. Gunung Sewu in Prehistoric Times. Yogyakarta: Gadja Mada University Press; 2002.

24. Simanjuntak T, Asikin N. Early Holocene human settlement in eastern Java. Bull Indo-Pac Prehistory Assoc. 2004;24:13–9.

25. Détroit F. *Homo sapiens* in Southeast Asian Archipelagos: The Holocene fossil evidence with special reference to funerary practices in East Java. In: Simanjutak T, Pojoh IHE, Hisyam M, editors. Austronesian diaspora and the ethnogeneses of people in Indonesian Archipelago Proceedings of the International Symposium. Indonesian Institute of Sciences. International Center for Prehistoric and Austronesian Studies.; 2006.

26. Pawlik A, Crozier R, Fuentes R, Wood R, Piper P. Burial traditions in early Mid-Holocene Island Southeast Asia: new evidence from Bubog-1, Ilin Island, Mindoro Occidental. Antiquity. 2019;93(370):901–18.

27. Noerwidi S. Using dental metrical analysis to determine the Terminal Pleistocene and Holocene population of Java. In: Piper PJ, Matsumura H, Bulbeck D, editors. New perspectives in Southeast Asia and Pacific prehistory. Canberra: ANU Press; 2017. p. 79–96. (Terra Australis).

28. Yondri L. Kubur prasejarah temuan dari Gua Pawon desa gunung masigit, kabutapen Bandung provinsini Jawa barat: Sumbangan data bagi kehidupan prasejarah di sekitar tepian danau Bandung purba. [Jakarta]: Universitas Indonesia; 2005.

29. Barker G, Lloyd-Smith L, Barton H, Cole F, Hunt C, Piper PJ, et al. Foraging-farming transitions at the Niah caves, Sarawak, Borneo. Antiquity. 2011;85(328):492–509.

30. Barker G, Lloyd-Smith L. The prehistoric funerary archaeology of the niah caves, sarawak (malaysian borneo). In: Moyes H, editor. Sacred darkness: A global perspective on the ritual use of caves. University Press Boulder, Colorado, USA; 2012. p. 249–62.

31. Lloyd-Smith L. Early Holocene burial practice at Niah cave, Sarawak. Bull Indo-Pac Prehistory Assoc. 2012;32:54–69.

32. Lara M, Paz V, Lewis H, Solheim II W. Bone modifications in an early Holocene cremation burial from Palawan, Philippines. Int J Osteoarchaeol. 2015;25:637–52.

33. Piper PJ. Human cultural, technological and adaptative changes from the end of the Pleistocene to the mid-Holocene in Southeast Asia. In: Oxenham M, Buckley HR, editors. The Routledge Handbook of Bioarchaeology in Southeast Asia and the Pacific Islands. Routledge; 2016. p. 24–44.

34. Lloyd-Smith L, Barker G, Barton H, Cameron J, Cole F, Daly P, et al. “Neolithic” societies c.4000-2000 years ago: Austronesian farmers? In: Barker G, editor. Rainforest foraging and farming in Island Southeast Asia. Cambridge, UK: McDonald Institute for Archaeological Research; 2013. p. 255–98. (The Archaeology of Niah Cave, Sarawak; vol. 1).

35. Arifin K. Early human occupation of the east Kalimantan rainforest. [Canberra]: Australian National University; 2004.

36. Carlhoff S, Duli A, Nägele K, Nur M, Skov L, Sumantri I, et al. Genome of a middle Holocene hunter-gatherer from Wallacea. Nature. 2021 Aug 26;596(7873):543–7.

37. Bulbeck D, Aziz FA, O'Connor S, Calo A, Fenner JN, Marwick B, et al. Mortuary caves and the Dammar trade in the Towuti-Routa region, Sulawesi, in an Island Southeast Asian context. Asian Perspect. 2016;55(2):148–83.

38. Lewis H, Paz V, Lara M, Barton H, Piper P, Ochoa J, et al. Terminal Pleistocene to mid-Holocene occupation and an early cremation burial at Ille Cave, Palawan, Philippines. Antiquity. 2008;82:318–35.

39. Bulbeck D. Bioarchaeological analysis of the Northern Moluccan excavated human remains. In: Bellwood P, editor. The Spice Islands in Prehistory: Archaeology in the Northern Moluccas, Indonesia. ANU Press; 2019. p. 167–99. (Terra Australis).

40. Bulbeck D. The Last Glacial Maximum human burial from Liang Lembudu in Northern Sahulland. In: O'Connor S, Spriggs M, Veth P, editors. The Archaeology of the Aru Islands, Eastern Indonesia. ANU E Press; 2006. p. 255–94. (Terra Australis).

41. Bulbeck D. Human remains from Liang Nabulei Lisa, Aru Islands. In: O'Connor S, Spriggs M, Veth P, editors. The Archaeology of the Aru Islands, Eastern Indonesia. Canberra: ANU E Press; 2006. p. 163–70.

42. Samper Carro SC, Stewart TJ, Mahirta, Wood R, O'Connor S. Burial practices in the early mid-Holocene of the Wallacean Islands: A sub-adult burial from Gua Makpan, Alor Island, Indonesia. Quat Int. 2021 Nov;603:125–38.

43. Galipaud J-C, Kinaston R, Halcrow S, Foster A, Harris N, Simanjuntak T, et al. The Pain Haka burial ground on Flores: Indonesian evidence for a shared neolithic belief system in Southeast Asia. Antiquity. 2016;90(354):1505–21.

44. Hawkins S, Arumdhati FS, Litster M, Lim TS, Basile G, Leclerc M, et al. Metal-age maritime culture at jareng bori rockshelter, pantar island, eastern indonesia. Rec Aust Mus. 2020;72:237–62.
