# Supplementary material for: Talking Dead. New burials from Tron Bon Lei (Alor Island, Indonesia) inform on the evolution of mortuary practices from the terminal Pleistocene to the Holocene in Southeast Asia
Source: PLoS One. 2022 Aug 24;17(8):e0267635. doi: 10.1371/journal.pone.0267635 (PMC9401180; doi:10.1371/journal.pone.0267635)
Supplement: S2 File — Detailed description of the skeletal elements recovered in the three burials presented in this manuscript. (PDF) [file pone.0267635.s002.pdf]

S2 File. Description of skeletal elements from Tron Bon Lei

All the necessary permits for this study have been obtained according to the regulations. The material described was excavated under a RISTEK Foreign Research Permit granted by the Indonesian government (O'Connor 1304/FRP/SM/V/2014 & 3024/FRP/E5/Dit.KI/IX/2017). Following excavation, all the material recovered from Tron Bon Lei was transported to the Universitas Gadjah Mada (UGM; Yogyakarta, Indonesia). Upon signing of a Material Transfer Agreement for non-destructive analysis between the Universitas Gadjah Mada and the Australian National University (ANU), the material was imported under a permit to import biological products granted by the Australian Government, Department of Agriculture, Water and Environment (0004271052), and deposited at the quarantine accredited site (room 3.127) in Coombs Building (Department of Archaeology and Natural History, ANU), where is currently kept. The material will be handed over to the UGM (Archaeology Department) and stored with the archaeological material recovered from Tron Bon Lei upon publication of the results. No catalog accession numbers have yet been assigned to the material as it has not been deposited in a museum. The analysis of the remains followed the guidelines established in the Code of Ethics of the American Association of Physical Anthropologists (2003).

Anatomical description

Burial TLB-1

The high fragmentation of the vertebrae and ribs impeded their exact anatomical identification, although some of the vertebral remains have been assigned to the last thoracic vertebra and the lumbar segment (L-1 to L-4). Fragments of vertebral bodies and processes identified might correspond to portions of the thoracic segment. A small portion of the corpus sterni was identified.

The left humerus is represented by the distal diaphysis, preserved until 4cm above the olecranon fossa, and the complete distal epiphysis. The spiral breakage outline indicates that fragmentation happened shortly after deposition (i.e. fresh bone breakage). The diaphysis was recovered in 2014, as

well as fragments of trabecular bone that were likely part of the proximal epiphysis. The left radius and ulna are complete, but fragmented in three portions longitudinally.

The right humerus was recorded during the excavation of the skeletal remains (Fig. 10), but this element was not documented during the laboratory tasks. Nevertheless, some fragments of cortical bone recorded in the laboratory could correspond with the fragmented diaphysis, although neither of the epiphyses were clearly identified. The right radius and ulna are practically complete, broken in several fragments. The ulna preserves the trochlear notch, with the posterior part of the olecranon missing. Both hands are partially represented, with the anatomical identification of some of the elements uncertain due to fragmentation or being unable to observe the complete elements due to connection of elements through calcareous coating (see *3.3.5 Articulation assessment*).

The pelvic girdle is heavily fragmented. Both ossa coxae are represented by large portions of the ilium, included the greater sciatic notch and auricular surfaces, and the acetabuli (Fig. X). A portion of the right pubis was identified, preserving the pubic tubercle, a fragment of the obturator crest, and the anterior part of the symphyseal surface. The sacrum was highly fragmented and covered in calcareous coating.

The right femur and tibia are almost complete, with both epiphyses present in the femur and only the distal epiphysis in the tibia, although the three elements are fragmented. The right fibula represented by small fragments of the cortical surfaces, averaging 4cm in length and a quarter of the original circumference. The right patella is highly fragmented, preserving the lateral posterior surface.

The left femur is almost complete, with most of the proximal epiphysis and the complete diaphysis preserved, with the lesser trochanter absent. The distal epiphysis is broken in several fragments. The left tibia is complete but missing the medial part of the distal epiphysis and with a fragmented proximal epiphysis. Despite fragmentation, this is the only element that was available to estimate the individual stature (see *Bioskeletal profile*). The shaft of the left fibula is complete, with both epiphyses broken. The left patella is complete and with a good preservation.

Both feet are represented by most of the skeletal elements, only missing some of the phalanges.

Burial DE B2

The cranial skeleton is represented by a partial right maxilla and fragmented mandible. Several fragments were identified as likely to be part of the cranial vault, although the poor preservation of the remains made their anatomical identification impractical. The fragment of right maxilla preserved the portion where it joins to the zygomatic process. The right upper dentition preserved included the first and second premolars, and the first and second molars.

The left upper dentition was represented by isolated teeth, including the first premolar, and first and second molar. Some isolated roots, likely to correspond to the incisors, canine and second premolar were recorded, although the extreme dental wear observed (i.e. apical part of the root preserved) hampered its anatomical identification.

The mandible was broken into three fragments, with two conjoining fragments from the left side. The right portion of the mandible preserved the third molar, with evidence of antemortem loss of the second molar (i.e. obliterated alveolus) The most anterior part of the left mandible corresponds to the mandibular foramen portion, with the first and second premolar preserved. The conjoining fragment shows evidence of antemortem loss of the first and second molars, with the third molar preserved in place.

The vertebral column is represented by fragments of the atlas, vertebral bodies, lamina and spinous processes from the cervical vertebrae (3rd to 7th), and one fragment of transverse process assigned the fifth lumbar vertebra.

Several ribs were recovered, although their high fragmentation impeded their anatomical identification. The preserved portion of the ribs comprise mainly small fragments of the rib shaft (ranging from 1 to 4 cm), with a few fragments preserving the sternal end.

Between a quarter and half of the right scapula is preserved. This element preserves a fragmented glenoid fossa, the scapular neck and the lateral border, without coracoid process. The blade preserves mostly its lateral portion, with several isolated fragments of the blade recorded. The left scapula preserves a larger portion of the glenoid fossa, as well as a fragmented acromion, the scapular neck and the lateral border. Several fragments of the blade were documented.

The right humerus is fragmented in several portions, some of them preserving around half of the original circumference and with evidence of post-depositional collapse of the diaphysis. The largest fragment identified (around half of the original length) preserved the anteromedial and posterior distal surfaces, and the lateral and medial borders. A fragment of the proximal epiphysis was identified, fused, preserving the greater tubercle, most of humeral head (with the antero-medial portion missing) and the anatomical neck. The left humerus is more fragmented than the right, represented by small fragments of diaphysis. A crest or ridge was identified in one of the fragments, tentatively identified as the medial border.

No fragments of the right zygopodia were identified, while both left radius and ulna were recovered. The left radius is fragmented in two, with one of the portions preserving the distal epiphysis. The epiphysis preserves the ulnar notch and the anterior side, with the lateral and posterior surfaces broken. The two fragments identified from the left ulna preserve their complete original circumference, with the interosseous border, ulnar tuberosity and supinator crest preserved.

More elements from the right hand are preserved compared with the left hand. The right hand is represented by the lunate, capitate, the complete metacarpal series and several proximal, intermediate and distal phalanges. The left hand is represented by the lunate, capitate and hamate, partial second and fourth metacarpals, some proximal phalanges and the intermediate second phalanx.

The pelvic girdle is preserved in a fragmentary state. The right coxae is represented by a partial acetabulum, preserving the acetabular notch, acetabular margin, with a broken ischial spine, and a fragment of right pubis preserving the distal portion of the symphyseal surface and the lateral pubic body. The left coxae is represented by the acetabular margin and the greater sciatic notch. The coccyx preserves the second to fourth coccygeal vertebrae.

The right femur is extremely fragmented, only identified from fragments of cortical and cancellous bone. The left femur is broken in three large fragments which preserved their complete circumference. These three conjoining fragments comprise the distal portion of the femur, preserving a fragment of the medial condyle from the distal epiphysis, as well as the complete linea aspera, and the medial and supracondylar line.

The right tibia is as fragmented as the femur, identified by fragments of cortical and trabecular bone. Conversely, the left tibia is almost complete, missing both epiphyses but preserving its complete original circumference and most of its original length.

The right fibula is broken into three shaft fragments and an additional fragment preserving the malleolar articular surface. The left fibula is broken into four fragments, missing the distal epiphysis but with the proximal fibular articular surface preserved.

The right patella is almost complete, preserving the apex and the lateral articular surface.

The right foot is represented by the proximal portion of the second metatarsal, while the left foot is almost complete. Only the left calcaneus and talus were recovered.

Burial E16b

The cranial skeleton is represented by a partial cranial vault, and the upper dentition. The identified portion of the frontal bone comprises a fragment preserving the left temporal line and the supraorbital margin. A portion of the right parietal was reconstructed, including the occipital angle, portions of the lambdoidal suture and the parietal striae. The left parietal preserves a fragment of the sagittal suture and the parietal tuber, although both parietals could not be conjoined. The right temporal is fragmentary, preserving the sphenoid. The right mastoid, and the right petrous bone were recovered isolated. The occipital bone is represented by a right portion, which includes the lambdoidal suture and conjoins with the right parietal. Several fragments were recognised as belonging to the facial skeleton (nasal bones and maxillae), although their reconstruction was not possible due to the heavy fragmentation and fragility of the bones. The upper dentition is complete, but only the crowns are preserved due to brittle roots. The cervical line is preserved in only two of the right molars.

The postcranial skeleton is represented by axial and autopodia elements. The vertebral column is represented by a fragmentary cervical vertebral body (C-3), fragments of vertebral bodies assigned to the lowest thoracic vertebrae, and fragmented lumbar vertebrae (L-1, L2 and L-5). The sternum is represented by the top part of the corpus sterni. A large number of fragmented rib bodies was recovered, with the only ribs identified anatomically consisting of the right and left tenth rib and the right eleventh rib.

The pelvic girdle is represented by an almost complete coccyx, a fragmentary sacrum (preserving the proximal portion) and fragments of the os coxae: the right pelvis preserving the ilium and acetabulum, and the left pelvis represented by the pubis, the proximal border of the iliac crest and the distal border of the ischium.

The autopodia are well represented (Fig.4). The right carpals preserved are the trapezoid, trapezium, lunate and triquetral, while the pisiform is missing from the left carpal series. Both left and right metacarpal are partially represented, with both first metacarpals, and the right third metacarpal identified. Several proximal, intermediate and distal phalanges were recovered, mostly identified to the right side.

The lower limbs are represented by a right patella and incomplete feet. Both calcanei and astragali, as well as the complete tarsal series from both feet and some of the sesamoids, were recovered. Most of the metatarsals from both feet were recovered, as well as several proximal, intermediate and distal foot phalanges were identified from both feet (Fig. 4).

Heavy fragmentation of some elements, such as metapodials, vertebral and rib bodies, hampered their anatomical identification and siding. Additionally, some elements individually packed during excavation but not identified in situ, were too fragmented after transport to be identified in the laboratory.
